# Association between antihypertensive treatment and adverse events: systematic review and meta-analysis

**DOI:** 10.1136/bmj.n189

**Published:** 2021-02-10

**Authors:** Ali Albasri, Miriam Hattle, Constantinos Koshiaris, Anna Dunnigan, Ben Paxton, Sarah Emma Fox, Margaret Smith, Lucinda Archer, Brooke Levis, Rupert A Payne, Richard D Riley, Nia Roberts, Kym I E Snell, Sarah Lay-Flurrie, Juliet Usher-Smith, Richard Stevens, F D Richard Hobbs, Richard J McManus, James P Sheppard

**Affiliations:** 1 Nuffield Department of Primary Care Health Sciences, Radcliffe Primary Care Building, University of Oxford, Oxford, OX2 6GG, UK; 2 School of Medicine, Keele University, Keele, UK; 3 Oxford University Hospitals NHS Foundation Trust, Oxford, UK; 4 Primary Care Unit, Department of Public Health and Primary Care, University of Cambridge, Cambridge, UK; 5 NIHR Oxford Biomedical Research Centre, Oxford University Hospitals NHS Foundation Trust, Oxford, UK; 6 Centre for Academic Primary Care, Population Health Sciences, University of Bristol, Bristol, UK; 7 Bodleian Health Care Libraries, University of Oxford, Oxford, UK

## Abstract

**Objective:**

To examine the association between antihypertensive treatment and specific adverse events.

**Design:**

Systematic review and meta-analysis.

**Eligibility criteria:**

Randomised controlled trials of adults receiving antihypertensives compared with placebo or no treatment, more antihypertensive drugs compared with fewer antihypertensive drugs, or higher blood pressure targets compared with lower targets. To avoid small early phase trials, studies were required to have at least 650 patient years of follow-up.

**Information sources:**

Searches were conducted in Embase, Medline, CENTRAL, and the Science Citation Index databases from inception until 14 April 2020.

**Main outcome measures:**

The primary outcome was falls during trial follow-up. Secondary outcomes were acute kidney injury, fractures, gout, hyperkalaemia, hypokalaemia, hypotension, and syncope. Additional outcomes related to death and major cardiovascular events were extracted. Risk of bias was assessed using the Cochrane risk of bias tool, and random effects meta-analysis was used to pool rate ratios, odds ratios, and hazard ratios across studies, allowing for between study heterogeneity (τ^2^).

**Results:**

Of 15 023 articles screened for inclusion, 58 randomised controlled trials were identified, including 280 638 participants followed up for a median of 3 (interquartile range 2-4) years. Most of the trials (n=40, 69%) had a low risk of bias. Among seven trials reporting data for falls, no evidence was found of an association with antihypertensive treatment (summary risk ratio 1.05, 95% confidence interval 0.89 to 1.24, τ^2^=0.009). Antihypertensives were associated with an increased risk of acute kidney injury (1.18, 95% confidence interval 1.01 to 1.39, τ^2^=0.037, n=15), hyperkalaemia (1.89, 1.56 to 2.30, τ^2^=0.122, n=26), hypotension (1.97, 1.67 to 2.32, τ^2^=0.132, n=35), and syncope (1.28, 1.03 to 1.59, τ^2^=0.050, n=16). The heterogeneity between studies assessing acute kidney injury and hyperkalaemia events was reduced when focusing on drugs that affect the renin angiotensin-aldosterone system. Results were robust to sensitivity analyses focusing on adverse events leading to withdrawal from each trial. Antihypertensive treatment was associated with a reduced risk of all cause mortality, cardiovascular death, and stroke, but not of myocardial infarction.

**Conclusions:**

This meta-analysis found no evidence to suggest that antihypertensive treatment is associated with falls but found evidence of an association with mild (hyperkalaemia, hypotension) and severe adverse events (acute kidney injury, syncope). These data could be used to inform shared decision making between doctors and patients about initiation and continuation of antihypertensive treatment, especially in patients at high risk of harm because of previous adverse events or poor renal function.

**Registration:**

PROSPERO CRD42018116860.

## Introduction

High blood pressure (hypertension) is one of the leading modifiable risk factors for cardiovascular disease worldwide,^1^ and much healthcare resource is given to reducing blood pressure. In recent years, guidelines for hypertension management have recommended lower treatment targets^2^ ^3^ on the basis of trials that found benefit for cardiovascular risk reduction.^4^ In patients with frailty and multimorbidity, however, these guidelines recommend clinical judgment because of potential risks from adverse effects of treatment.^3^ ^5^

In the UK, guidelines for managing patients with multimorbidity suggest doctors weigh the risk of diseases with the benefits and risks of treatments and make personalised treatment recommendations.^6^ Such an approach is straightforward for the benefits of treatment when data exist from numerous meta-analyses of randomised controlled trials.^7^ ^8^ ^9^ When attempting to judge the potential harms of treatment, however, few data are available to support decision making. Existing meta-analyses focus on the overall risk of adverse events,^10^ ^11^ making it difficult to distinguish between those events that might not be considered particularly serious, such as transient electrolyte abnormalities, and those resulting in severe complications and hospital admission, such as falls or acute kidney injury.

Currently few definitive data are available from meta-analyses of randomised controlled trials on the risks of specific harm outcomes that could be used to facilitate personalised decision making in patients with hypertension. We systematically reviewed evidence from trials and large observational studies to determine the association between antihypertensive treatment and specific adverse events such as falls, acute kidney injury, and electrolyte abnormalities.

## Methods

We performed a systematic review and meta-analysis of randomised controlled trials and large observational studies examining the association between antihypertensive treatment and adverse events. The study is reported according to the preferred reporting items for systematic reviews and meta-analyses (PRISMA) guidelines.^12^ The study protocol was registered on PROSPERO (international prospective register of systematic reviews) and is available online (www.crd.york.ac.uk/prospero , CRD42018116860).

### Search strategy

To capture all randomised controlled trials reporting the association between antihypertensive treatment and adverse events we searched Embase(OvidSP), Medline(OvidSP), Cochrane Central Register of Controlled Trials (CENTRAL, Cochrane Library), and the Science Citation Index (Web of Science Core Collection). Searches were undertaken from inception of the databases until 14 April 2020, and no language restrictions were applied. In this review we focused on randomised controlled trials, which are less prone to bias from confounding by indication.^13^ ^14^ We also searched for large observational studies by interrogating the bibliographies of databases of electronic health records, but as few relevant data were identified and given the limitations of observational study designs we decided not to include them in the present study. Further studies were identified through searching the references of eligible full text articles and previous meta-analyses. Supplementary table 1 shows the full search strategy.

### Selection of studies and inclusion and exclusion criteria

Eligible studies included participants aged 18 years or older, compared individuals receiving antihypertensive treatment (single agents) with those receiving placebo or no treatment, more antihypertensive drugs compared with fewer antihypertensive drugs, or one blood pressure target compared with another. Although these study designs examine different types of intervention, all compared more antihypertensive treatment with less antihypertensive treatment, enabling the potential association with adverse events to be determined. Trials were also required to present data describing the association between antihypertensive treatment and at least one adverse event. Randomised controlled trials were included if they reported 50 or more adverse events in each specific category or had at least 650 patient years of follow-up.

To ensure study selection and data analysis remained manageable by avoiding small, early phase mechanistic studies, we specified a priori the limit on patient years of follow-up and number of outcome events. We chose the specific criteria to ensure each included study was large enough to accrue outcome events and provide reliable effect estimates. These criteria assumed an incidence of the primary outcome (falls) of 7.8 events per 100 patient years of follow-up, which would accrue at least 50 outcome events in each study.^15^

We excluded studies in specialist populations (children, pregnant women), and case reports, case series, or before and after studies. At least two members of the review team (AA, MS, BP, SF, CK, AD, JPS) independently reviewed study titles, abstracts, and full text articles. At each stage, the entire review team screened a proportion of articles to ensure consistency of decision making. Disagreements were resolved by a third reviewer (JPS).

### Outcome measures

Outcomes of interest were prespecified based on those reported in recent large scale trials of blood pressure lowering treatment.^4^ ^16^ ^17^ The primary outcome was falls, at any time point and by any definition given in the original study. Secondary outcomes were acute kidney injury, fractures, gout, electrolyte abnormalities (changes in potassium), hypotension, and syncope (eg, fainting) at any time point during trial follow-up. Acute kidney injury was defined as any outcome reported according to the KDIGO (kidney disease: improving global outcomes) definition.^18^ All other outcomes were defined according to definitions given in the original study. Additional treatment efficacy outcomes of interest included cardiovascular death, myocardial infarction, stroke, and all cause mortality.

### Data extraction and quality assessment

AA, MH, LA, AD, and BL extracted data from eligible studies. Two reviewers independently entered outcome data into a Microsoft Excel spreadsheet (2016 version, Redmond, WA). A second reviewer then manually cross checked these, referring to the original source data when discrepancies were identified. After an initial consistency check involving extraction of data from 10 articles, one reviewer extracted study descriptive data.

Data were extracted on populations studied, interventions tested, length of follow-up, effect measures (estimates and confidence intervals for rate ratios, odds ratios, and hazard ratios), and numbers of patients experiencing adverse events and cardiovascular or mortality outcomes.

The methodological quality and risk of bias of individual studies was assessed using the Cochrane risk of bias tool (for randomised controlled trials).^19^

### Data synthesis

Summary effect estimates describing the association between all antihypertensive drug classes (combined) and adverse events were derived using a random effects meta-analysis. For uncommon adverse events (approximately less than 10% of the population experience an event), rate ratios (for rate outcomes), odds ratios (for binary outcomes), and hazard ratios (for time-to-event outcomes) were considered reasonably similar and combined provided they had the same directional interpretation.^20^ For uncommon outcomes, we label summary effect estimates as risk ratios. For more common cardiovascular disease outcomes, we synthesised rate ratios, odds ratios, and hazard ratios separately. We used restricted maximum likelihood estimation to fit the random effects model, with 95% confidence intervals derived using the Hartung-Knapp approach to account for uncertainty in heterogeneity estimates.^21^ For studies with three treatment arms, we split binary and rate outcomes for the control arm into two equal groups.^22^ This approach is not possible for the time-to-event outcomes, and therefore we made an approximate adjustment to the standard errors.

Heterogeneity was summarised using the estimate of between study variance (τ^2^) and 95% prediction intervals for the treatment effect in a new study. The proportion of variability in effect estimates due to between study heterogeneity was summarised using I^2^.

Sensitivity analyses were undertaken focusing on adverse events reported as a reason for study withdrawal. Meta-regression was used to examine the association between observed treatment effects and study quality. Small study effects (potential publication bias) were explored using contour enhanced funnel plots for outcomes reported in 10 or more studies.^23^ Prespecified subgroup analyses were conducted to examine the association between treatment and adverse events by antihypertensive drug class.

No other subgroup analyses were undertaken by patient level characteristics (eg, age), owing to the risk of ecological bias.^24^ Aggregate data only allow relationships across studies to be examined, but these often do not reflect within study (participant level) relationships, because of aggregation bias and study level confounding.^25^ ^26^ For example, those studies with a higher mean age might also have a longer mean follow-up or a higher dose of the drug; hence it is difficult to disentangle these different associations, and interpreting across study associations as if they were interactions at the individual level is potentially misleading.

All analyses were undertaken using Stata version 16 (StataCorp, College Station, TX).

### Patient and public involvement

This study was developed with the help of our patient and public advisor. As a member of our study advisory group, they commented on the study protocol. We also held a focus group with seven older adults during the study to discuss broader issues related to drugs for cardiovascular disease prevention and adverse events, which informed the interpretation of this work.

## Results

### Study selection and characteristics

**Fig 1:**
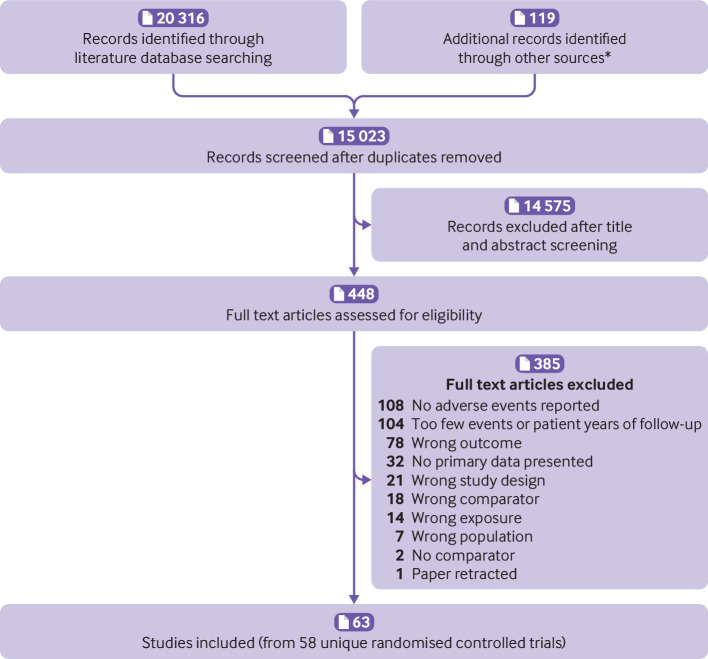
Selection of studies for inclusion in review. *Hand searches of reference lists of included studies and recent meta-analyses of blood pressure lowering trials^7^ ^8^ ^9^

A total of 15 023‬ unique articles were identified from the literature searches, of which 119 records were screened from reference lists of included articles and previous meta-analyses. After screening of the title, abstract, and full text, 63 articles originating from 58 randomised controlled trials^4^ ^16^ ^27^ ^28^ ^29^ ^30^ ^31^ ^32^ ^33^ ^34^ ^35^ ^36^ ^37^ ^38^ ^39^ ^40^ ^41^ ^42^ ^43^ ^44^ ^45^ ^46^ ^47^ ^48^ ^49^ ^50^ ^51^ ^52^ ^53^ ^54^ ^55^ ^56^ ^57^ ^58^ ^59^ ^60^ ^61^ ^62^ ^63^ ^64^ ^65^ ^66^ ^67^ ^68^ ^69^ ^70^ ^71^ ^72^ ^73^ ^74^ ^75^ ^76^ ^77^ ^78^ ^79^ ^80^ ^81^ ^82^ ^83^ ^84^ ^85^ ^86^ ^87^ were eligible for inclusion (fig 1). The most common reason for exclusion at full text screening was lack of adverse event reporting (n=108) or inclusion of too few patient years of follow-up (n=104).

A total of 280 638 participants were included in the primary analyses from 58 unique randomised controlled trials. Forty eight studies compared a single drug treatment with placebo and 10 studies compared a high blood pressure target with a lower blood pressure target in the intervention and control groups (table 1). The remaining five studies either compared treatment with no treatment or compared multiple drugs with a single drug. The median duration of follow-up in the trials was 3 (interquartile range 2-4) years. Most studies were conducted in patients with at least one risk factor for cardiovascular disease in addition to hypertension.

**Table 1:** Summary of included randomised controlled trials

Trial name, year, reference	Population characteristics	Total sample	Follow-up	Mean (SD) age (years): intervention; control	Mean (SD) baseline sBP (mm Hg): intervention: control	Intervention	Comparator
AASK 2002^37^	African-Americans with renal disease	1094	3.8 years	54.5 (10.9); 54.7 (10.4)	152 (25); 149 (23)	Mean atrial pressure target ≤92 mm Hg	Mean atrial pressure target 102-107 mm Hg
ACCORD 2010^16^ ^48^	Type 2 diabetes	4733	5.6 years	62.2 (6.8); 62 (6.9)	139.0 (16.1) 139.4 (15.5)	BP target <120 mm Hg	BP target <140 mm Hg
ACEi progressive renal insufficiency study group 1996^59^	Renal dysfunction	583	3 years	51 (13); 51 (12)	142 (17); 144 (17)	Benazepril	Placebo
ADVANCE 2014^70^	Type 2 diabetes	11 140	4.3 years	66 (6); 66 (7)	145 (22); 145 (21)	Perindopril+indapamide	Placebo
AIRE 1993^81^	Acute myocardial infarction+evidence of heart failure	1986	15 months	64.9 (10); 65.1 (10.8)	NS (28% hypertensive)	Ramipril	Placebo
ALTITUDE 2012^85^	Type 2 diabetes	8561	2.6 years	64.6 (9.6); 64.4 (9.9)	137.3 (16.2); 137.3 (16.7)	Aliskiren	Placebo
ASPIRE 2011^86^	Post-myocardial infarction	820	36 weeks	61 (12); 59 (12)	121.6 (16.1); 121.7 (16.2)	Aliskiren	Placebo
BEST 2001^87^	NYHA class III or IV heart failure	2708	2 years	60 (12.6); 60 (12.3)	117 (18.2); 117 (17.8)	Bucindolol	Placebo
BHAT 1982^27^	Admitted to hospital with acute myocardial infarction	3837	2 years	54.7 (NS); 54.9 (NS)	112.3 (NS); 111.7 (NS)	Propranolol	Placebo
Cardio-Sis 2009^28^	No diabetes with hypertension	1111	2 years	67 (7); 67 (7)	163.3 (11.3); 163.3 (11.1)	BP target <120 mm Hg	BP target <130 mm Hg
CCS-I 1997^29^	Acute myocardial infarction	14 962	4 weeks	61.2 (10.7); 61 (10.6)	127 (24); 126 (24)	Captopril	Placebo
CHARM -Preserved 2003^30^	NYHA class II-IV heart failure	3023	Median 36.6 months	67.2 (11.1); 67.1 (11.1)	136.0 (18.6); 136.3 (18.3)	Candesartan	Placebo
CHARM-ADDED 2003^31^	NYHA class II-IV heart failure	2548	3.5 years	64 (10.7); 64.1 (11.3)	124.7 (18.6); 125.6 (18.6)	Candesartan	Placebo
CHARM-Alternative 2003^32^	Heart failure	2028	2.7 years	66.3 (11); 66.8 (10.5)	129.9 (19.0); 130.3 (18.5)	Candesartan	Placebo
Collaborative Study Group 2001^33^	Type 2 diabetes with nephropathy	1715	2.6 years	59.3 (7.1), 59.7 (7.9); 58.3 (8.2)	160 (20); 159 (19); 158 (20)	Irbesartan or Amlodipine	Placebo
CONSENSUS II 1992^34^	Post-myocardial infarction	6090	6 months	65.7 (NS); 65.8 (NS)	133 (NS); 134 (NS)	Enalapril	Placebo
DIME 2014^35^	No diabetes, hypertension	1130	4.4 years	63 (10); 63 (10)	154 (11); 154 (10)	Thiazide diuretic	No thiazide diuretic
Dutch TIA Trial 1993^36^	Previous transient ischaemic attack	1473	2.6 years	50 (NS); 54 (NS)	157 (25) and 158 (24)	Atenolol	Placebo
EMPHASIS-HF 2011^38^	NYHA class II heart failure	2737	1.75 years	68.6 (7.7); 68.6 (7.6)	124 (17); 124 (17)	Eplerenone	Placebo
EUROPA 2003^39^	Stable coronary heart disease without heart failure	12 218	4.2 years	60 (9); 60 (9)	137 (16); 137 (15)	Perindopril	Placebo
EWPHE 1991^40^	>60 years with raised BP	822	5 years	72 (8); 72 (8)	183 (16); 183 (16)	Hydrochlorothiazide+triamterene	Placebo
GISSI-3 1994^41^	Myocardial infarction within 24 hours	9442	6 weeks	NS	NS	Lisinopril	No treatment
GISSI-AF 2009^42^	Atrial fibrillation and underlying CVD	1442	1 year	67.5 (9.5); 68.2 (8.9)	138.2 (16.7); 139.0 (16.9)	Valsartan	Placebo
Hypertension in diabetes study IV 1996^43^	Type 2 diabetes	758	5 years	57 (7.9) all patients	160 (19); 160 (20)	Atenolol or Captopril with BP target <150/<85 mm Hg	BP target <180/<105 mm Hg
HOPE Trial^44^ ^45^	>55 years, high CVD risk	9297‬‬‬‬‬‬‬‬‬‬‬‬‬‬‬‬‬‬‬‬‬‬‬‬‬‬‬‬‬‬‬‬‬‬‬‬‬‬‬‬‬‬‬‬‬‬‬‬‬‬‬‬‬‬‬‬‬‬‬	5 years	66 (7); 66 (7)	139 (20); 139 (20)	Ramipril	Placebo
HOPE-3 2016^46^	Men >55 years and women >65 years with one CVD risk factor or more	12 705	5.5 years	65.7 (6.4); 65.8 (6.4)	138.2 (14.7); 137.9 (14.8)	Candesartan+Hydrochlorothiazide	Placebo
HYVET Trial^47^ ^84^	>80 years with hypertension	3845	2.1 years	84/84	173 and 173	Indapamide and/or perindopril	Placebo
INFINITY 2019^49^	>75 years, hypertension, white matter lesions	199	3 years	80.9 (4.4); 80.3 (3.8)	149.7 (15.4); 152.0 (17.5)	sBP target ≤130 mm Hg	sBP target ≤145 mm Hg
Intensive Antihypertensive Treatment for Elderly 2013^50^	>70 years with hypertension	724	4 years	76.6 (4.6); 76.5 (4.5)	158.8 (16.0); 160.3 (16.9)	BP target <140/90 mm Hg	BP target <150/90 mm Hg
I-PRESERVE 2008^51^	Heart failure	4128	4.1 years	72 (7); 72 (7)	137 (15); 136 (15)	Irbesartan	Placebo
MACB 1995^52^	Referred for coronary artery bypass grafting	967	2 years	Median age 64 in both groups	Median sBP 120 mm Hg in both groups	Metoprolol	Placebo
MERIT-HF 2000^53^	NYHA class II-IV heart failure	3991	1 year	63.9 (NS); 63.7 (NS)	Not stated (44% of cohort hypertensive)	Metoprolol	Placebo
MRC 1985^55^	Patients with mild hypertension	17 354	5.5 years	51 (NS); 53 (NS)	158 (men); 165 (women)	Bendroflumethiazide or propranolol	Placebo
Multicentre Diltiazem Postinfarction Trial 1988^56^	Admitted to hospital with acute myocardial infarction	2466	25 months	58 (10); 58 (10)	NS	Diltiazem	Placebo
NAVIGATOR 2010^57^	Type 2 diabetes	9306	6.3 years	63.7 (6.8) 63.8 (6.8)	139.4 (17.8) and 139.9 (17.1)	Valsartan	Placebo
NICOLE 2003^58^	<75 years and previous successful angioplasty	819	3 years	60.4 (NS); 60.2 (NS)	NS (40% of cohort hypertensive)	Nisoldipine	Placebo
NILVAD 2018^60^	Alzheimer’s disease	511	1.5 years	73.1 (8.7); 72.8 (7.8)	138 (14); 137 (14)	Nilvadipine	Placebo
ONTARGET 2008^61^	Existing vascular disease or diabetes	25 620	4.5 years	66.4 (7.2) 66.4 (7.1) 66.5 (7.3)	141.8 (17.4); 141.7 (17.2); 141.9 (17.6)	Ramipril or telmisartan	Ramipril+telmisartan combination
ORIENT 2011^62^	Type 2 diabetes with poor renal function	566	3.4 years	59.1 (8.1)/; 59.2 (8.1)	141.7 (17.0) 140.8 (18.0)	Olmesartan	Placebo
PEACE 2004^63^	Myocardial infarction or bypass in past 3 months	8290	4.8 years	64 (8); 64 (8)	134 (17) and 133 (17)	Trandolapril	Placebo
PRoFESS 2008^64^	>55 years and ischaemic stroke	20 332	2.5 years	66.1 (8.6); 66.2 (8.6)	144.1 (16.4) 144.2 (16.7)	Telmisartan	Placebo
PROGRESS 2001^65^	Previous stroke or transient ischaemic attack	6105	4 years	64 (10); 64 (10)	147 (19); 147 (19)	Perindopril+indapamide	Placebo
ROADMAP Trial^66^ ^67^	Type 2 diabetes	4447	3.2 years	57.7 (8.8); 57.8 (8.6)	137 (16); 136 (15)	Olmesartan	Placebo
SANDS 2009^68^	Native Americans with type 2 diabetes	548	3 years	55.8 (9.3); 57.4 (9.3)	128.7 (14.7) 132.6 (16.4)	BP target <115/75 mm Hg	BP target <130/80 mm Hg
SENIORS 2005^69^	>70 years with heart failure	2128	1.5 years	76.1 (4.8); 76.1 (4.6)	138.6 (20.1) 139.5 (21.1)	Nebivolol	Placebo
SHEP 1991^71^ ^72^	>60 years with ISH	4736	5 years	71.6 (6.7); 71.5 (6.7)	170.5 (9.5) 170.1 (9.2)	Chlorthalidone with or without atenolol or reserpine	Placebo
SOLVD 1992^73^	Heart failure with ejection fraction <0.35	2569	41.4 months	60.7 (NS); 61.0 (NS)	125.3 (NS); 124.5 (NS)	Enalapril	Placebo
Spironolactone and mild heart failure 2016^74^	NYHA class II heart failure	139	10 years	66.7 (1.3); 65.5 (1.3)	120.6 (1.3); 121.3 (1.4)	Spironolactone+standard treatment	Standard treatment
SPRINT 2015^4^	>50 years with increased CVD risk, no diabetes	9361	3.26 years	67.9 (9.4); 67.9 (9.5)	139.7 (15.8); 139.7 (15.4)	BP target <120 mm Hg	BP target <140 mm Hg
SPS3 2013^75^	Stroke within past 6 months	3020	3.7 years	63 (11); 63 (11)	142 (19); 144 (19)	BP target <130 mm Hg	BP target 130-149 mm Hg
The Norwegian Multicenter Study 1981^76^	Admitted to hospital with acute myocardial infarction	1884	17 months	60.3 (NS); 61.4 (NS)	NS	Timolol	Placebo
TRACE 1995^86^	Admitted to hospital with acute myocardial infarction	1749	24 to 50 months	67.7 (NS); 67.3 (NS)	122 (NS); 120 (NS)	Trandolapril	Placebo
TRANSCEND 2008^77^	CVD or diabetes with end organ damage	5926	Median 56 months	66.9 (7.3); 66.9 (7.4)	140.7 (16.8); 141.3 (16.4)	Telmisartan	Placebo
TROPHY 2006^78^	Prehypertensive population	772	4 years	48.6 (7.9); 48.3 (8.2)	133.9 (4.3); 134.1 (4.2)	Candesartan	Placebo
VA NEPHRON-D 2013^79^	Type 2 diabetes+moderate to severe proteinuria	1448	2.2 years	64.5 (7.9); 64.7 (7.7)	136.9 (16.5); 137.0 (16.0)	Losartan+lisinopril	Losartan+placebo
Val-HeFT 2001^80^	Heart failure	5010	23 months	62.4 (11.1) 63.0 (11)	123.0 (18.4) 124.0 (18.6)	Valsartan	Placebo
VALIANT 2003^82^	Myocardial infarction with left ventricular systolic dysfunction	11 703	2 years	65.0 (11.8) 64.9 (11.8) 64.6 (11.9)	123 (NS) overall mean	Valsartan or captopril	Valsartan+captopril (dual treatment)
VA-NHLBI 1978^83^	21-50 years with mild hypertension	1012	2 year	37.5 (NS); 37.5 (NS)	Mean diastolic BP 93 mm Hg	Chlorthalidone and reserpine	Placebo

CVD=cardiovascular disease; NYHA=New York Heart Association; NS=not stated; sBP=systolic blood pressure; ISH=isolated systolic hypertension.

### Quality assessment

**Fig 2:**
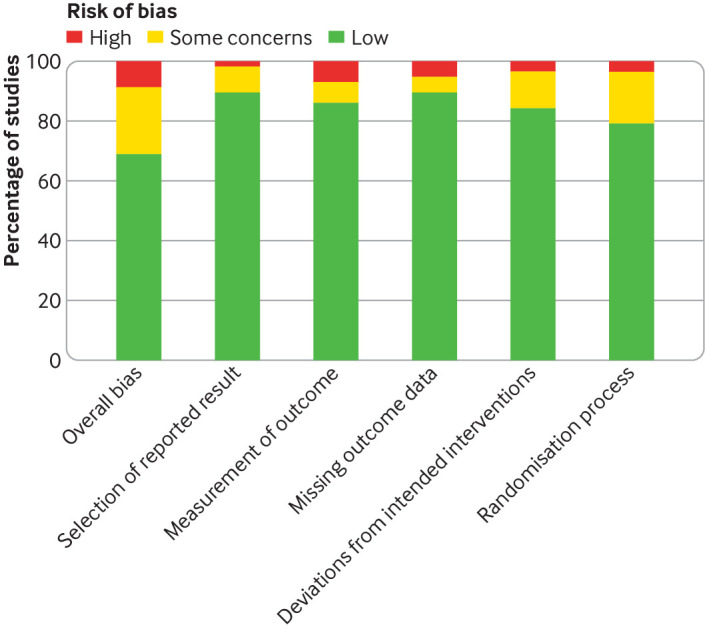
Summary of risk of bias assessment across all included randomised controlled trials

Supplementary table 2 presents the risk of bias assessment for individual trials. Most of the trials (n=40, 69%) had a low risk of bias (fig 2). Eight trials (14%) did not adequately blind outcome assessment of adverse events (or did not describe this adequately) and 12 (21%) did not adequately describe the randomisation process. Outcome reporting was complete in 52 trials (90%) trials.

### Primary outcome

**Fig 3:**
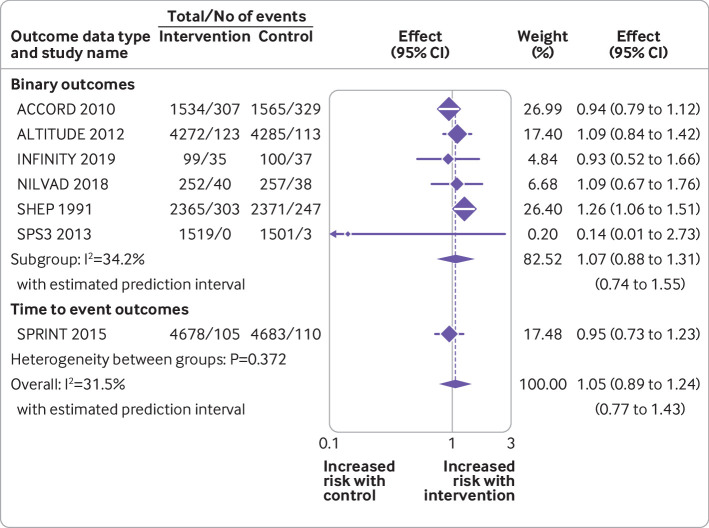
Random effects meta-analysis of randomised controlled trials examining the association between antihypertensive treatment and falls

Seven randomised controlled trials reported data for the primary outcome of falls (fig 3). Data were available from 29 481‬ patients experiencing 1790 events. Overall, no evidence was found of an association between antihypertensive treatment and falls (summary risk ratio 1.05, 95% confidence interval 0.89 to 1.24). Little evidence was found of between study heterogeneity in this association (τ^2^=0.009; I^2^=31.5%; P=0.372). Subgroup analyses by drug type did not reveal any evidence of associations between falls and specific antihypertensive drug classes, except for thiazide diuretics, although this was based on data from just one trial (supplementary figure 1).^71^ More intensive treatment (ie, to lower blood pressure targets) was not associated with falls across four trials (supplementary figure 1).

### Secondary outcomes

**Fig 4:**
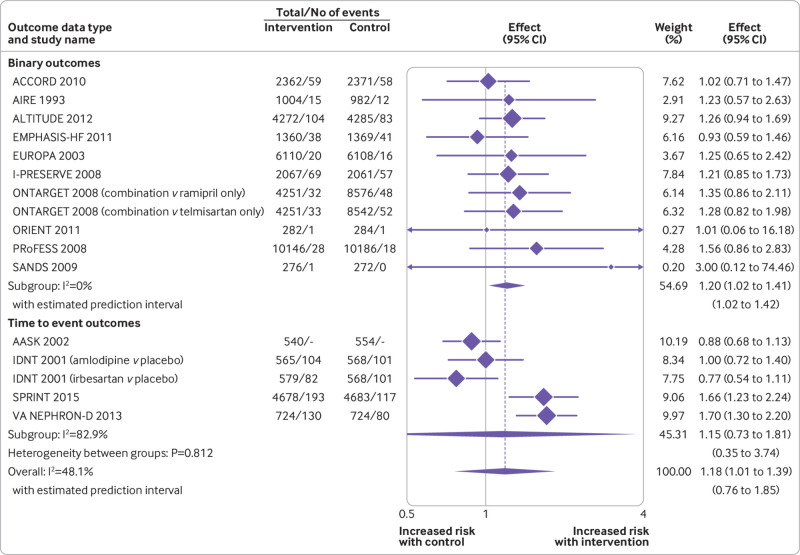
Random effects meta-analysis of randomised controlled trials examining the association between antihypertensive treatment and acute kidney injury

In analyses examining adverse events across all drug classes, antihypertensive treatment was associated with an increased risk of acute kidney injury (summary risk ratio 1.18, 95% confidence interval 1.01 to 1.39, n=15 studies; fig 4), hyperkalaemia (1.89, 1.56 to 2.30, n=26 studies), hypotension (1.97, 1.67 to 2.32, n=35 studies), and syncope (1.28, 1.03 to 1.59, n=16 studies) (table 2; supplementary figures 2-4), although statistical heterogeneity was significant for most outcomes (τ^2^=0.037 to 1.374; I^2^=42.9% to 85.1%). Evidence was unclear of an association between antihypertensive treatment and fractures (0.93, 0.58 to 1.48, τ^2^=0.062, I^2^=53.8%, n=5 studies; supplementary figure 5) and gout (1.54, 0.63 to 3.75, τ^2^=1.612, I^2^=94.3%, n=12 studies; supplementary figure 7), although confidence intervals were wide, partly reflecting large between study heterogeneity.

**Table 2:** Main analyses showing meta-analysis results from trials reporting the association between antihypertensive treatment and adverse events and cardiovascular and mortality outcomes

Outcome	No of studies	Sample size			Events		Effect size (95% CI)*	I^2^ (%)	τ^2^	95% prediction interval
		Intervention group	Control group		Intervention group	Control group				
**Adverse events**										
Falls^4^ ^16^ ^49^ ^60^ ^71^ ^72^ ^75^ ^85^ (primary outcome)	7	14 719	14 762		913	877	1.05 (0.89 to 1.24)	31.5	0.009	0.77 to 1.43
Acute kidney injury^4^ ^16^ ^33^ ^38^ ^39^ ^51^ ^61^ ^62^ ^64^ ^68^ ^79^ ^81^ ^85^ ^88^	15	43 467	52 133		909	785	1.18 (1.01 to 1.39)	48.1	0.037	0.76 to 1.85
Fractures^16^ ^50^ ^60^ ^71^ ^72^ ^89^	5	6447	6466		230	267	0.93 (0.58 to 1.48)	53.8	0.062	0.36 to 2.41
Gout^17^ ^35^ ^55^ ^83^ ^90^	5	16 524	16 137		249	26	3.84 (0.95 to 15.57)	84.3	1.374	0.11 to 138.91
Hyperkalaemia^4^ ^16^ ^30^-^34^ ^38^ ^41^ ^43^ ^45^ ^51^ ^57^ ^59^ ^62^ ^64^ ^68^ ^73^ ^74^ ^77^ ^79^ ^82^ ^85^ ^86^ ^91^-^93^	26	57 604	61 795		2749	1880	1.89 (1.56 to 2.30)	71.8	0.121	0.90 to 3.98
Hypokalaemia^4^ ^16^ ^35^ ^38^ ^43^ ^51^ ^57^ ^71^ ^72^ ^74^ ^83^ ^86^ ^94^	12	19 748	19 528		517	274	1.54 (0.63 to 3.75)	94.3	1.612	0.08 to 29.98
Hypotension^4^ ^16^ ^27^ ^29^-^32^ ^34^ ^36^ ^38^ ^39^ ^42^ ^51^-^53^ ^56^ ^58^ ^62^ ^64^ ^65^ ^68^-^70^ ^75^ ^76^ ^78^ ^80^-^82^ ^85^-^87^ ^91^ ^93^	35	88 575	93 547		5390	3121	1.97 (1.67 to 2.32)	85.1	0.132	0.92 to 4.18
Syncope^4^ ^16^ ^17^ ^27^ ^60^ ^61^ ^63^ ^64^ ^68^ ^75^-^78^ ^81^ ^85^ ^87^	16	51 072	51 189‬		644	543	1.28 (1.03 to 1.59)	42.9	0.050	0.75 to 2.17
**Cardiovascular and mortality outcomes**										
All cause mortality^4^ ^16^ ^17^ ^28^ ^31^ ^32^ ^34^ ^36^ ^38^ ^42^ ^56^ ^57^ ^62^ ^63^ ^69^ ^71^ ^72^ ^74^ ^75^ ^77^ ^79^-^81^ ^85^-^87^ ^89^ ^91^	32	128 619‬	128 729		11 831	13 018	0.93 (0.88 to 0.98)	50.4	0.008	0.77 to 1.12
Cardiovascular death^4^ ^16^ ^17^ ^30^-^32^ ^36^ ^45^ ^51^ ^57^ ^61^-^63^ ^69^ ^71^ ^72^ ^75^ ^77^ ^82^ ^85^ ^87^ ^91^ ^92^	21	92 676	92 733		6341	6890‬	0.92 (0.86 to 0.99)	54.6	0.011	0.73 to 1.16
Myocardial infarction^4^ ^16^ ^17^ ^28^ ^32^ ^38^ ^45^ ^57^ ^61^-^63^ ^71^ ^72^ ^75^ ^77^ ^79^ ^85^ ^87^ ^89^ ^91^ ^92^	19	75 002‬	75 301		2900	3255	0.94 (0.85 to 1.03)	40.7	0.013	0.73 to 1.21
Stroke^4^ ^16^ ^17^ ^28^ ^36^ ^38^ ^45^ ^57^ ^61^-^64^ ^75^ ^77^ ^79^ ^85^ ^89^ ^92^	17	104 153	104 366		3220	3733	0.84 (0.76 to 0.93)	44.8	0.013	0.64 to 1.09

* Adverse events reported as risk ratios and cardiovascular and mortality outcomes reported as hazard ratios (in studies reporting outcome as time to event). Binary and rate outcomes for cardiovascular and mortality outcomes are presented in supplementary figures 15-17.

Analyses of outcomes by specific drug class showed that drugs affecting the renin angiotensin-aldosterone system were associated with acute kidney injury (1.26, 1.03 to 1.56, τ^2^=0.030, I^2^=39.0%; n=9 studies; table 3, supplementary figure 8) and hyperkalaemia (2.03, 1.67 to 2.48, τ^2^=0.063, I^2^=51.0%; n=20 studies; table 3, supplementary figure 9). These effects were larger and had less between study heterogeneity than in analyses examining the association between all antihypertensive treatments and the same outcomes (table 2 and table 3). Only a small number of studies assessed the association between diuretics and hypokalaemia (three studies) or gout (five studies), and the results of these were inconclusive (table 3; supplementary figures 10 and 11). No other drug class specific associations with adverse events were observed in the stratified analyses (supplementary figures 12-14).

**Table 3:** Summary of sensitivity analyses showing important drug class specific associations between antihypertensive treatment and specific adverse events

Outcome	Drug class	No of studies	Sample size			Events		Risk ratio (95% CI)	I^2^ (%)	τ^2^	95% prediction interval
			Intervention group	Control group		Intervention group	Control group				
Acute kidney injury^33^ ^39^ ^51^ ^61^ ^62^ ^64^ ^79^ ^81^ ^85^	RAAS	9	33 686	42 316		514	468	1.26 (1.03 to1.56)	39.0	0.030	0.80 to 1.99
Hyperkalaemia^30^-^34^ ^42^ ^45^ ^51^ ^57^ ^59^ ^62^ ^64^ ^73^ ^77^ ^79^ ^82^ ^85^ ^86^ ^91^-^93^	RAAS	20	47 122	51 787		2282	1541	2.03 (1.67 to 2.48)	51.0	0.063	1.16 to 3.57
Hypokalaemia^35^ ^71^ ^72^ ^83^	Diuretics	3	3154	3114		259	25	10.73 (0.32 to 354.58)	80.9	1.385	-
Gout^17^ ^35^ ^55^ ^71^ ^72^ ^83^ ^90^	Diuretics	5	12 121	12 190		237	29	4.48 (0.79 to 26.54)	85.0	1.547	0.05 to 388.68

RAAS=drugs affecting the renin angiotensin-aldosterone system (eg, angiotensin converting enzyme inhibitors, angiotensin II receptor blockers, direct renin inhibitors); diuretics=thiazide and thiazide-like diuretics.

Other generic adverse events such as falls, hypotension, syncope, and fractures were examined by drug class, but no significant drug specific effects were observed (supplementary figures 1 and 12-14).

### Cardiovascular and mortality outcomes

**Fig 5:**
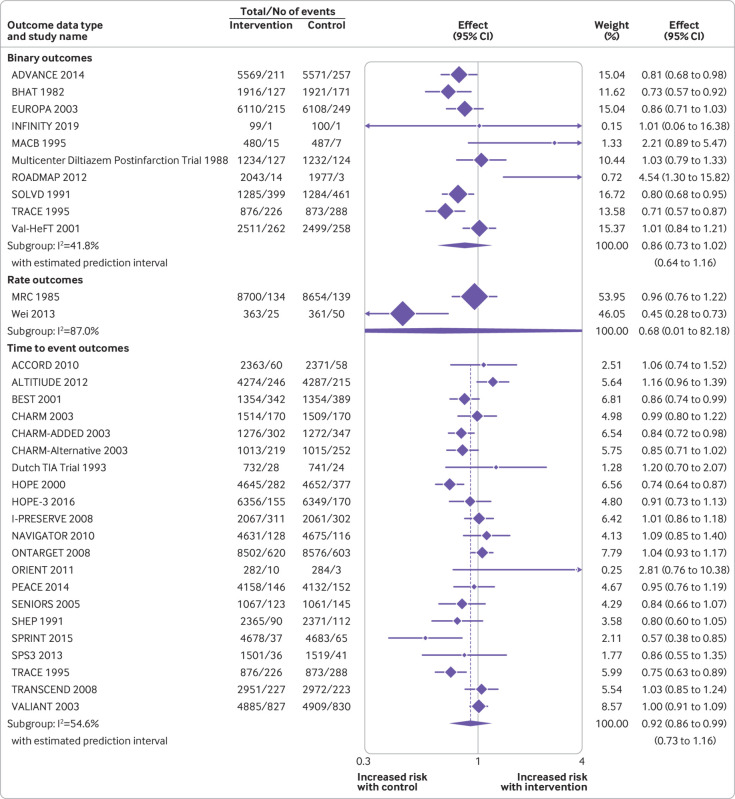
Random effects meta-analysis of randomised controlled trials examining the association between antihypertensive treatment and cardiovascular death

On average across studies examining outcomes using time-to-event analyses, antihypertensive treatment was associated with a reduction in cardiovascular death (hazard ratio 0.92, 95% confidence interval 0.86 to 0.99, τ^2^=0.011, I^2^=54.6%, n=21 studies; fig 5), all cause mortality (0.93, 0.88 to 0.98, τ^2^=0.008, I^2^=50.4%, n=32 studies; supplementary figure 15), and stroke (0.84, 0.76 to 0.93, τ^2^=0.013, I^2^=44.8%, n=17; supplementary figure 16) (table 2). No clear evidence was found of an association between antihypertensive treatment and myocardial infarction (supplementary figure 17).

### Sensitivity analyses

Meta-regression examining the relation between the observed treatment effects for each adverse event outcome and study quality found no clear evidence of an association (supplementary table 3). Funnel plots showed asymmetry (potential publication bias) for hyperkalaemia and hypotension events, with smaller studies missing for smaller effect estimates, but this was not evident for other adverse events examined (supplementary figures 18-22).

Supplementary figures 23-27 show the results of sensitivity analyses focusing on studies reporting adverse events that led to participant withdrawal from each trial (summarised in table 4). These analyses were limited to studies reporting acute kidney injury, gout, hyperkalaemia, hypotension, and syncope owing to availability of data. In these analyses, summary risk ratios for hyperkalaemia, hypotension, and syncope were increased compared with the primary analysis including all studies. However, there was no longer evidence that acute kidney injury was associated with antihypertensive treatment (table 4).

**Table 4:** Sensitivity analyses showing meta-analysis results focusing on trials reporting the association between antihypertensive treatment and adverse events which led to permanent withdrawal from a trial

Outcome	No of studies	Sample size			Events		Risk ratio (95% CI)	I^2^ (%)	τ^2^	95% prediction interval
		Intervention group	Control group		Intervention group	Control group				
Acute kidney injury^38^ ^39^ ^61^ ^62^ ^64^ ^85^	6	30 672	39 350		128	146	1.34 (0.99 to 1.81)	0.0	0.000	0.97 to 1.84
Gout^17^ ^55^	2	15 998	15 959		179	38	3.41 (0.08 to 148.47)	86.4	1.903	-
Hyperkalaemia^30^-^34^ ^38^ ^42^ ^45^ ^59^ ^62^ ^64^ ^82^ ^85^ ^92^	13	34 580	38 953		398	189	2.28 (1.70 to 3.05)	22.8	0.053	1.28 to 4.06
Hypotension^27^ ^30^-^32^ ^34^ ^38^ ^39^ ^42^ ^52^ ^53^ ^62^ ^64^ ^65^ ^70^ ^76^ ^80^ ^82^ ^85^	18	51 063	56 030		1042	541	2.18 (1.84 to 2.58)	34.0	0.033	1.43 to 3.32
Syncope^17^ ^27^ ^61^ ^64^ ^77^ ^85^	6	34 146	34 289		62	28	2.17 (1.20 to 3.90)	0.0	0.000	1.15 to 4.09

## Discussion

Data from random effects meta-analyses of 58 randomised controlled trials and more than 280 000 patients with hypertension confirm the known benefit of antihypertensive treatment in reducing the risk of cardiovascular disease.^7^ ^8^ ^9^ These data also confirm the association between antihypertensive treatment and adverse events^10^ ^11^ and show how this association varies across some drug classes and for mild (eg, hypotension without falls) and more severe (eg, acute kidney injury, syncope) adverse events. Despite a widely held belief,^95^ ^96^ no association was found between treatment and falls, but an association with syncope was observed, which is important as this can have a major impact on quality of life and health service use and could even result in death.^97^ ^98^ ^99^ ^100^

These data will inform shared decision making around initiation and continuation of antihypertensive treatment, especially in patients with a high absolute risk of certain adverse outcomes as a result of previous events or poor renal function. Such discussions will become increasingly important as patients age and develop frailty and multimorbidity that could put them at increased risk of adverse events.^101^ ^102^ ^103^

### Strengths and limitations of this study

More than 15 000 articles were screened for inclusion in this review and 58 randomised controlled trials including a large number of participants and adverse events were identified. Although power was likely to be sufficient to detect associations between antihypertensive treatment and adverse events, we observed statistically significant heterogeneity across studies, and the resulting prediction intervals were wide. Such heterogeneity might preclude pooling of some treatment effects, so caution should be exercised when interpreting the results. For acute kidney injury and hyperkalaemia events, the observed heterogeneity was partly explained by pooling of different drug classes, and heterogeneity was reduced when we focused on drugs that affect the renin angiotensin-aldosterone system. For other outcomes, the observed heterogeneity could not be explained by study quality or differences in the drug class examined in individual trials; however, populations of interest, interventions, comparators, and study designs varied widely across studies, which could have contributed to the observed variation.

As this review focused on adverse events, selective outcome reporting might also have been a problem. Evidence was found of publication bias for certain outcomes (hyperkalaemia and hypotension), confirming the findings of previous studies that showed adverse events are more likely to be reported in randomised controlled trials when they are statistically significant.^104^ This is understandable in the context of single trial reporting, but it would be better for the evidence base if all adverse events were reported in clinical trials to enable more complete meta-analyses in the future. It is a limitation of this review that original study authors were not contacted for these additional data.

This review focused on large randomised controlled trials with the aim of including those with at least 50 adverse events (and therefore 650 patient years of follow-up). This restriction on study size was chosen to make the review more manageable in terms of screening and analysis and avoid inclusion of numerous small early phase mechanistic studies of varying methodological quality. The cut-off for this inclusion was chosen to ensure studies provided adequately powered estimates of association between treatment and outcomes.^15^ It is possible that some useful trials could have been excluded, although many relevant trials were still available for inclusion.

Across all included trials, adverse events were poorly defined and probably varied across studies. For instance, many studies referred to syncope as an outcome, but did not say what type of syncopal event this might have included. A conservative approach to inclusion of outcomes was taken when possible, and only those explicitly stating the outcome of interest were included. For example, trials reporting hypotension or acute kidney injury were included, but those reporting hypotension or dizziness or renal impairment were excluded. Despite this approach, some studies were included that did not specify the thresholds used to define hypotension or acute kidney injury. This could have resulted in some relevant data for certain outcomes being missed, but this meant those that were included were likely to be sufficiently similar to enable pooling in a meta-analysis. Although the quality of adverse event ascertainment is likely to have varied between trials, it would not be expected to vary between treatment arms within trials. Thus it is unlikely that differences in the quality of adverse event ascertainment would have affected the relative treatment effects presented in this review.

We prespecified adverse events of interest based on those reported in recent large scale trials of blood pressure lowering treatment.^4^ ^16^ ^17^ Other patient focused harm outcomes, such as weight gain, sexual dysfunction, fatigue, and exercise intolerance might exist that were reported in the original trials but not captured as part of this review. However, the reporting of these events is likely to vary because many have no standardised definitions.^105^ ^106^ ^107^ Some might be captured but not reported.^108^ It is also important to note that randomised controlled trials often select populations with less frailty and multimorbidity who are more likely to tolerate treatment.^109^ Therefore, fewer adverse events might have been reported in the included trials than would be expected in the general population.

For outcomes included in meta-analyses, the time points at which they occurred varied across studies, and so the risk ratios and odds ratios provided relate to a summary across different times. We did synthesise hazard ratios when available, but these were rarely reported.

### Comparison with other studies

Few previous meta-analyses have quantified the association between antihypertensive treatment and adverse events. Thomopoulos and colleagues examined the association between antihypertensive treatment and permanent discontinuation of treatment because of adverse events and found that antihypertensives were associated with a near doubling of risk (standardised relative risk 1.89, 95% confidence interval 1.51 to 2.39).^10^ ^110^ This was similar to findings from our sensitivity analyses focusing on permanent withdrawal as a result of hyperkalaemia, hypotension, and syncope events. These associations were stronger than those observed in the primary analysis focusing on all adverse event reporting. It is possible that these events were more likely to be reported in the intervention group when they were considered serious enough to lead to withdrawal.^104^ Although the focus of this review was on adverse events, we found evidence for the beneficial effects of treatment on all cause mortality, cardiovascular mortality, and stroke, but not on myocardial infarction, as has been reported previously.^4^ ^8^

Frey and colleagues^11^ focused on data from seven original studies investigating the harms of intensive blood pressure lowering targets (≤130 mm Hg) versus usual care (<140 mm Hg). Although this number of studies was insufficient to conduct a meta-analysis, the descriptive summary suggested that intensive blood pressure lowering might be associated with higher rates of serious adverse events. The present analysis included all trials of blood pressure lowering treatment enabling meta-analyses of the association between antihypertensive treatment and adverse events and how this association varies across mild and more severe adverse events. We identified an increased risk of acute kidney injury, hyperkalaemia, hypotension, and syncope with antihypertensive treatment.

Stratified analyses by drug class suggested that associations with acute kidney injury and hyperkalaemia were mostly driven by the use of drugs that affect the renin angiotensin-aldosterone system (eg, angiotensin converting enzyme inhibitors, angiotensin II receptor blockers, and direct renin inhibitors). However, no evidence was found of an association with this class of drug and falls, fractures, gout, or hypokalaemia. In analyses that focused on patients prescribed diuretics, a 10-fold increase in the risk of hypokalaemia was observed, but this association was derived from only three trials, with high between study heterogeneity. The pooled effect had large confidence intervals and was not statistically significant. This null finding contrasted with previous studies that recommend routine monitoring of potassium to detect hypokalaemia in patients prescribed diuretics.^111^ This could be explained by the small number of included trials examining this drug class.

Much debate exists in the literature on the association between antihypertensive treatment and falls.^95^ ^96^ ^112^ ^113^ Most data showing an association originate from observational studies,^112^ ^113^ which are prone to bias from confounding by indication.^14^ Despite conflicting evidence, a wide held belief remains that antihypertensive treatment increases the risk of falls.^95^ ^96^ This study found no evidence for an association between treatment or lower blood pressure targets and falls, but an association was found with syncope. Although syncope is a common cause of falls, not all falls are caused by syncope and therefore not all falls will be related to blood pressure lowering treatment.^114^ In addition, reporting of falls might vary among participants (ie, not all participants will be admitted to hospital or see their primary care doctor after a fall) and participants might be more likely to be withdrawn from a trial when experiencing events that could be considered precursors to falls and fractures (eg, hypotension). If this were the case and hypotension events are not dealt with by treating doctors, the incidence of serious falls and fractures associated with antihypertensive treatment could be greater in routine clinical practice.

### Policy implications

The present data clearly show the benefits and harms of antihypertensive treatment for specific cardiovascular outcomes and adverse events. The data also highlight that certain adverse events might be specific to certain drug classes (eg, renin angiotensin-aldosterone system drugs and acute kidney injury or hyperkalaemia). This detail is important because some adverse events reported in randomised controlled trials might be considered relatively mild and worth the risk when weighed against the substantial benefits of treatment. These new data will allow patients and clinicians to take into consideration these benefits and risks, as has been recommended in clinical guidelines.^6^ This is particularly important now that guidelines for the management of hypertension across the world increasingly recommend more intensive treatment,^2^ ^3^ ^5^ ^115^ but with conflicting blood pressure targets, meaning a personalised approach is required for each patient.

The present data should ideally be combined with information about an individual’s absolute risk of each harm outcome to make informed, personalised treatment decisions. This process is complex and requires real time data, which suggests that tools embedded in electronic health records will be the way forward. Further work is needed to understand better the results of this meta-analysis (which summarises average risk ratios across all participants and studies) in the context of individualised absolute risks so that treatment initiation and discontinuation can be targeted at those with the most to gain.^116^ In the absence of such information, doctors should focus on patients who have experienced previous adverse events or have poor renal function.^17^ ^110^ ^117^

### Conclusions

This review found no evidence of an association between antihypertensive treatment and falls (primary outcome) or fractures but did show a variation in the association between antihypertensive treatment and mild (eg, hypotension without falls) and more severe (eg, acute kidney injury, syncope) adverse events. Some effects were found to be specific to the drug class used. In patients at high risk of drug harms because of previous adverse events or poor renal function, these data should be used to inform shared decision making between doctors and patients around initiation and continuation of antihypertensive treatment.

**What is already known on this topic**

Many meta-analyses exist of randomised controlled trials that examine the efficacy of antihypertensive treatment, but few have studied potential harms
Existing meta-analyses have focused on the association between antihypertensive treatment and all adverse events, grouping mild and more serious outcomes
The association between antihypertensive treatment and specific adverse events is unclear

**What this study adds**

In a meta-analysis of 58 randomised controlled trials, including 280 638 participants, no evidence was found of an association between antihypertensive treatment and falls (primary outcome) or fractures
Evidence was, however, found of an association between antihypertensive treatment and potentially both mild (hypotension) and more severe (acute kidney injury, syncope) adverse events
These data might be used to inform shared decision making between doctors and patients about the benefits and harms of initiation and continuation of antihypertensives, especially in those at high risk of harm because of previous adverse events or poor renal function

## Web extra

Extra material supplied by authors

## Notes

Contributors: JPS conceived the study and wrote the protocol with FDRH, RJM, RS, and RR. NR did the literature searches. AA, MS, BP, SF, CK, AD, and JPS screened articles for inclusion. MH, AA, LA, AD, and BL extracted data for analysis. MH undertook the meta-analysis and produced forest plots and summary results, under supervision of RR. AA and JPS wrote the first draft of the manuscript. All authors revised the manuscript and approved the final version. JPS is the guarantor for this work and accepts full responsibility for the conduct of the study, had access to the data, and controlled the decision to publish. The corresponding author (JPS) attests that all listed authors meet authorship criteria and that no others meeting the criteria have been omitted.

Ethical approval: Not required.

The manuscript’s guarantor (JPS) affirms that the manuscript is an honest, accurate, and transparent account of the study being reported; that no important aspects of the study have been omitted; and that any discrepancies from the study as originally planned (and, if relevant, registered) have been explained.

Data sharing: Requests for data sharing should be sent to the corresponding author at james.sheppard@phc.ox.ac.uk.

Dissemination to participants and related patient and public communities: No participants were included in this work. The findings of this work, including a lay summary of the results, will be made available on the study website (www.phc.ox.ac.uk/research/stratified-treatments/studies/stratifying-treatments-in-the-multi-morbid-frail-elderly-stratify-antihypertensives).

Provenance and peer review: Not commissioned; externally peer reviewed.
