# Supplementary material for: Association between antihypertensive treatment and adverse events: systematic review and meta-analysis
Source: BMJ. 2021 Feb 10;372:n189. doi: 10.1136/bmj.n189 (PMC7873715; doi:10.1136/bmj.n189)
Supplement: Supplementary file 1 — Supplementary information: additional tables 1-3 and figures 1-27 [file alba062412.ww.pdf]

The association between antihypertensive treatment and adverse events: a systematic review and meta-analysis of 58 randomised controlled trials including 280,638 patients with hypertension

Supplementary material

Ali Albasri, Miriam Hattle, Constantinos Koshiaris, Anna Dunnigan, Ben Paxton, Sarah Emma Fox, Margaret Smith, Lucinda Archer, Brooke Levis, Rupert A Payne, Richard D Riley, Nia Roberts, Kym IE Snell, Sarah Lay-Flurrie, Juliet Usher-Smith, Richard Stevens, FD Richard Hobbs, Richard J McManus, James P Sheppard *on behalf of the STRATIFY investigators*

Contents

1. **Supplementary table 1.** Medline search strategy

2. **Supplementary table 2.** Risk of bias assessment for included trials

3. **Supplementary figure 1.** Random effects meta-analysis of randomised controlled trials examining the association between antihypertensive treatment and falls by drug class

4. **Supplementary figure 2.** Random effects meta-analysis of randomised controlled trials examining the association between antihypertensive treatment and hyperkalaemia

5. **Supplementary figure 3.** Random effects meta-analysis of randomised controlled trials examining the association between antihypertensive treatment and hypotension

6. **Supplementary figure 4.** Random effects meta-analysis of randomised controlled trials examining the association between antihypertensive treatment and syncope

7. **Supplementary figure 5.** Random effects meta-analysis of randomised controlled trials examining the association between antihypertensive treatment and fractures

8. **Supplementary figure 6.** Random effects meta-analysis of randomised controlled trials examining the association between antihypertensive treatment and gout

9. **Supplementary figure 7.** Random effects meta-analysis of randomised controlled trials examining the association between antihypertensive treatment and hypokalaemia

10. **Supplementary figure 8.** Random effects meta-analysis of randomised controlled trials examining the association between antihypertensive treatment and acute kidney injury by drug class

11. **Supplementary figure 9.** Random effects meta-analysis of randomised controlled trials examining the association between antihypertensive treatment and hyperkalaemia by drug class

12. **Supplementary figure 10.** Random effects meta-analysis of randomised controlled trials examining the association between antihypertensive treatment and gout by drug class

13. **Supplementary figure 11.** Random effects meta-analysis of randomised controlled trials examining the association between antihypertensive treatment and hypokalaemia by drug class

14. **Supplementary figure 12.** Random effects meta-analysis of randomised controlled trials examining the association between antihypertensive treatment and fracture by drug class

15. **Supplementary figure 13.** Random effects meta-analysis of randomised controlled trials examining the association between antihypertensive treatment and hypotension by drug class

16. **Supplementary figure 14.** Random effects meta-analysis of randomised controlled trials examining the association between antihypertensive treatment and syncope by drug class

17. **Supplementary figure 15.** Random effects meta-analysis of randomised controlled trials examining the association between antihypertensive treatment and all-cause mortality

18. **Supplementary figure 16.** Random effects meta-analysis of randomised controlled trials examining the association between antihypertensive treatment and stroke

19. **Supplementary figure 17.** Random effects meta-analysis of randomised controlled trials examining the association between antihypertensive treatment and myocardial infarction

20. **Supplementary table 3.** Meta-regression examining the relationship between the observed treatment effects for each adverse event outcome and study quality

21. **Supplementary figure 18.** Funnel plots showing publication bias in studies reporting acute kidney injury outcomes

22. **Supplementary figure 19.** Funnel plots showing publication bias in studies reporting hyperkalaemia outcomes

23. **Supplementary figure 20.** Funnel plots showing publication bias in studies reporting hypokalaemia outcomes

24. **Supplementary figure 21.** Funnel plots showing publication bias in studies reporting hypotension outcomes

25. **Supplementary figure 22.** Funnel plots showing publication bias in studies reporting syncope outcomes

26. **Supplementary figure 23.** Random effects meta-analysis of randomised controlled trials examining the association between antihypertensive treatment and acute kidney injury leading to permanent withdrawal from each trial

27. **Supplementary figure 24.** Random effects meta-analysis of randomised controlled trials examining the association between antihypertensive treatment and gout leading to permanent withdrawal from each trial

28. **Supplementary figure 25.** Random effects meta-analysis of randomised controlled trials examining the association between antihypertensive treatment and hyperkalaemia leading to permanent withdrawal from each trial

29. **Supplementary figure 26.** Random effects meta-analysis of randomised controlled trials examining the association between antihypertensive treatment and hypotension leading to permanent withdrawal from each trial

30. **Supplementary figure 27.** Random effects meta-analysis of randomised controlled trials examining the association between antihypertensive treatment and syncope leading to permanent withdrawal from each trial

Supplementary table 1. Medline search strategy

# ▲	Search terms
1	hypertension/ or hypertension, malignant/ or hypertension, renal/ or hypertension, renovascular/
2	(hypertens* or antihypertens* or anti-hypertens* or blood pressure).ti.
3	*Blood Pressure/
4	*diastole/ or *systole/
5	(BP or DBP or SBP).ti,ab.
6	((diastol* or systol* or arterial) adj3 (pressure* or bp or mmHg or mmHg)).ti,ab.
7	1 or 2 or 3 or 4 or 5 or 6
8	antihypertensive agents/ or acebutolol/ or alprenolol/ or amlodipine/ or atenolol/ or bendroflumethiazide/ or bepridil/ or betaxolol/ or bethanidine/ or bisoprolol/ or bupranolol/ or captopril/ or carteolol/ or celiprolol/ or chlorisondamine/ or chlorothiazide/ or chlorthalidone/ or cilazapril/ or clonidine/ or cyclopenthiazide/ or diazoxide/ or dihydralazine/ or diltiazem/ or doxazosin/ or enalapril/ or enalaprilat/ or felodipine/ or fosinopril/ or guanabenz/ or hydralazine/ or hydrochlorothiazide/ or hydroflumethiazide/ or indapamide/ or indoramin/ or isradipine/ or labetalol/ or lisinopril/ or losartan/ or methyldopa/ or metipranolol/ or metolazone/ or metoprolol/ or mibefradil/ or minoxidil/ or nadolol/ or nicardipine/ or nimodipine/ or nisoldipine/ or nitrendipine/ or oxprenolol/ or pempidine/ or penbutolol/ or perindopril/ or pinacidil/ or pindolol/ or polythiazide/ or prazosin/ or propranolol/ or ramipril/ or timolol/ or todralazine/ or trichlormethiazide/ or xipamide/ or (antihypertensive or anti-hypertensive).ti,ab.
9	adrenergic alpha-antagonists/ or adrenergic alpha-1 receptor antagonists/ or doxazosin/ or indoramin/ or labetalol/ or prazosin/ or adrenergic alpha-2 receptor antagonists/ or adrenergic beta-antagonists/ or alprenolol/ or bupranolol/ or carteolol/ or dihydroalprenolol/ or metipranolol/ or nadolol/ or oxprenolol/ or penbutolol/ or pindolol/ or propranolol/ or sotalol/ or timolol/ or adrenergic beta-1 receptor antagonists/ or acebutolol/ or atenolol/ or betaxolol/ or bisoprolol/ or celiprolol/ or metoprolol/ or adrenergic beta-2 receptor antagonists/ or adrenergic beta-3 receptor antagonists/ or (adrenergic alpha-antagonist* or adrenergic alphaantagonist*).ti,ab.
10	angiotensin-converting enzyme inhibitors/ or captopril/ or cilazapril/ or enalapril/ or enalaprilat/ or fosinopril/ or lisinopril/ or perindopril/ or ramipril/ or angiotensin-converting enzyme inhibitor*.ti,ab.
11	angiotensin receptor antagonists/ or angiotensin ii type 1 receptor blockers/ or losartan/ or saralasin/ or angiotensin ii type 2 receptor blockers/ or angiotensin receptor antagonist*.ti,ab.
12	calcium channel blockers/ or amlodipine/ or amrinone/ or bencyclane/ or bepridil/ or diltiazem/ or felodipine/ or fendiline/ or flunarizine/ or gallopamil/ or isradipine/ or lidoflazine/ or mibefradil/ or nicardipine/ or nifedipine/ or nimodipine/ or nisoldipine/ or nitrendipine/ or tiapamil hydrochloride/ or verapamil/ or calcium channel blocker*.ti,ab.
13	diuretics/ or amiloride/ or bendroflumethiazide/ or chlorthalidone/ or cyclopenthiazide/ or furosemide/ or hydrochlorothiazide/ or hydroflumethiazide/ or indapamide/ or mefruside/ or methazolamide/ or methyclothiazide/ or metolazone/ or polythiazide/ or spironolactone/ or ticrynafen/ or triamterene/ or trichlormethiazide/ or xipamide/ or diuretics, osmotic/ or diuretics, potassium sparing/ or epithelial sodium channel blockers/ or mineralocorticoid receptor antagonists/ or sodium chloride symporter inhibitors/ or sodium potassium chloride symporter inhibitors/ or diuretic*.ti,ab.

14	vasodilator agents/ or bencyclane/ or bepridil/ or celiprolol/ or chromonar/ or colforsin/ or cromakalim/ or cyclandelate/ or diazoxide/ or dilazep/ or diltiazem/ or enoximone/ or ergoloid mesylates/ or erythritol/ or flunarizine/ or iloprost/ or isradipine/ or lidoflazine/ or minoxidil/ or molsidomine/ or nicardipine/ or nicergoline/ or nifedipine/ or nimodipine/ or nisoldipine/ or nitrendipine/ or nylidrin/ or oxprenolol/ or oxyfedrine/ or perhexiline/ or phenoxybenzamine/ or pinacidil/ or pindolol/ or prenylamine/ or s-nitrosoglutathione/ or suloctidil/ or trapidil/ or trimetazidine/ or verapamil/ or vasodilator*.ti,ab.
15	Aldosterone/
16	Chlorisondamine/
17	Mineralocorticoids/ or Desoxycorticosterone/ or Desoxycorticosterone Acetate/
18	Pempidine/
19	Renin-Angiotensin System/
20	(accupro or accuretic or acebutolol or acepril or acezide or adalat or adanif or adcirca or adipine or adizem or aldomet or aliskiren or alphavase or ambrisentan or amias or amiloride or amlodipine or amlostin or angilol or angiopine or angiozem or angitil or antipressan or apresoline or aprinox or aprovel or apsolox or atenamin or atenix or atenolol or baratol or baycaron or bedranol or bendroflumethiazide or benthiazide or berkatens or berkolol or berkozide or beta-adalat or beta-cardone or betadur or betaloc or beta-progane or bi-carzem or bipranix or bisoprolol or blocadren or bosentan or brevibloc or britazim or cabren or calanif or calazem or calchan or calcicard or calcilat or candesartan or capoten or capozide or capto-co or captomex or captopril or captopril or caracace or carace or cardene or cardicor or cardide or cardilate or cardioplen or cardozin or cardura or carvedilol or cascor or catapres or celectol or celiprolol or centyl or chlorothaizide or chlortalidone or cilazapril or ciolixil or clonidine or clopamide or co-amilozide or coaprovel or co-betaloc or co-diovan or congescor or co-prenozide or coracten or cordilox or corgard or corgaretic or coroday or co-tenidone or co-tenidone or coversyl or cozaar or co-zidocapt or cyclopenthiazide or cyclopenthiazide or delvas or diazoxide or dibenyline or dilcardia or diltiazem or dilzem or diovan or disogram or diurexan or diuril or dopamet or doxadura or doxazosin or ecopace or ednyt or emcor or enalapril or enalpril or enduron or eprosartan or esidrex or esmolol or ethibide or ethimil or eucardic or exforge or felendil or felodipine or felogen or felotens or fortipine or fosinopril or genalat or gopten or horizem or hydralazine or hydrenox or hydrochlorothiazide or hydroflumethiazide or hydromet or hygroton or hypertene or hypolar or hypovase or hytrin or iloprost or imidapril or indapamide or inderal or indipam or indoramin or innovace or innozide or irbesartan or isradipine or istin or kalspare or kalten or kaplon or keloc or kenzem or labetalol or labrocol or lacidipine or larbex or lercanidipine or lisinopril or lisopress or loniten or lopace or lopranol or lopresor or lopresoretic or lopresoteric or losartan or mapemid or mefruside or mepranix or meprobamate or metalpha or metenix or methyclothiazide or methyldopa or metolazone or metoprolol or metoros or metroprolol or micardis or minoxidil or moducren or moduretic or moexipril or monocor or motens or moxonidine or nadolol or natramid or natrilix or navidrex or nebivolol or neo-bendromax or neofel or neo-naclex or neozipine or nephril or nicardipine or nifedipine or nifedipress or nifelease or nimodrel or nimotop or nindaxa or nivaten or normetic or noyada or odrik or olmesartan or olmetec or optil or opumide or oxprenolol or parmid or perdix or perindopril or perinodopril or phenoxybenamine or phentolamine or physiotens or pindolol or pinodolod or plendil or pralenal or prazosin or prescal or prestim or probeta or propanix or propranolol or quinapril or quinil or ramipril or ranvera or raporsin or rapranol or rasilez or rawel or retalzem or revatio or rogitine or saluric or secadrex or sectral or securon or sevikar or sildenafil or slocinx or slofedipine or slo-pro or slopronol or slow-pren or slozem or sodium nitroprusside or sotacor or sotalol

	or spiro-co spironolactone or spiroprop or staril or syprol or tadalafil or tanatril or tarka or telmisartan or tenavoid or tenben or tenchlor or tenif or tenoret or tenoretic or tenormin or tensaid or tensipine or tensopril or terazosin or teveten or tildiem or timolol or tolerzide or totamol or totaretic or tracleer or trandate or trandolapril or trasicor or trasidrex or triamterene or triapin or tritace or uard or unipine or univer or valasartan or valni or varbim or vasaten or vascace or vascalpha or ventavis or verapamil or verapress or vera-til or vertab or viazem or visken or vivacor or xipamide or xuret or zanidip or zaroxolyn or zemret or zemtard or zestoretic or zestril or zida-co zildil or zolvera).mp.
21	8 or 9 or 10 or 11 or 12 or 13 or 14 or 15 or 16 or 17 or 18 or 19 or 20
22	exp Kidney Diseases/ or exp Kidney Failure, Chronic/
23	hypotension/
24	syncope/
25	electrolyte abnormalities/ or hypercalcemia/ or hypomagnesemia/ or hypophosphatemia/ or hyperkalemia/ or hypokalemia/ or hyponatremia/ or hyperglycemia/
26	gout/
27	accidental falls/
28	exp fractures, bone/
29	(((kidney or renal) adj2 (injur* or failure or insufficienc*)) or aki).ti,ab.
30	(falls or fracture? or syncope).ti,ab.
31	(electrolyte? or hypercalc* or hypomagnes* or hypophosphat* or hyperkal* or hypokal* or hyponatr* or hypoglyc*).ti,ab.
32	hypotensi*.ti,ab.
33	gout.ti,ab.
34	22 or 23 or 24 or 25 or 26 or 27 or 28 or 29 or 30 or 31 or 32 or 33
35	7 and 21 and 34
36	randomized controlled trial.pt.
37	controlled clinical trial.pt.
38	randomized.ab.
39	placebo.ab.
40	drug therapy.fs.
41	randomly.ab.
42	trial.ab.
43	groups.ab.
44	36 or 37 or 38 or 39 or 40 or 41 or 42 or 43
45	exp animals/ not humans.sh.
46	44 not 45
47	35 and 46
48	(case reports or comment or editorial or letter or news or "review").pt.
49	47 not 48

Supplementary table 2. Risk of bias assessment for included trials

Study	Randomization process	Deviations from intended interventions	Missing outcome data	Measurement of the outcome	Selection of the reported result	Overall Bias
AASK 2002	+	+	+	+	+	+
ACCORD 2010	+	+	+	+	+	+
ACEi progressive renal insufficiency study 1996	+	–	+	+	+	–
ADVANCE 2014	?	+	+	+	+	?
AIRE 1993	+	+	+	+	+	+
ALTITUDE 2012	+	+	+	?	+	+
ASPIRE 2011	+	+	+	+	+	+
BEST 2001	+	+	+	+	+	+
BHAT 1982	–	?	+	–	+	–
CARDIO-SIS 2009	+	+	+	+	?	?
CCS-I 1997	+	+	+	+	+	+
CHARM-ADDED 2003	+	+	?	+	+	?
CHARM-ALTERNATIVE 2003	+	+	+	+	+	+
CHARM-PRESERVED 2003	+	+	+	+	+	+
Collaborative Study group 2001	+	+	?	+	?	?
CONSENSUS II 1992	+	+	+	+	?	+
DIME 2014	–	?	+	–	+	–
Dutch TIA trial 1993	+	+	+	+	?	+
EMPHASIS-HF 2011	+	+	+	+	+	+
EUROPA 2003	+	+	+	+	+	+

EWPHE 1991	+	+	+	–	?	–
GISSI-3 1994	+	+	+	+	+	+
GISSI-AF 2009	?	+	+	+	+	?
HOPE 2007	+	+	+	+	+	+
HOPE-3 2016	+	+	+	+	+	+
Hypertension in Diabetes Study IV 1996	+	+	+	+	+	+
HYVET 2010	+	+	+	+	+	+
INFINITY 2019	+	+	+	+	+	+
Intensive Antihypertensive Treatment for Elderly 2013	?	+	+	?	+	?
I-PRESERVE 2008	+	+	+	+	+	+
MACB 1995	+	+	?	+	+	?
MERIT-HF 2000	+	?	–	+	+	?
MRC 1982	+	+	+	+	+	+
Multicenter Diltiazem Postinfarction Trial 1988	+	+	+	+	+	+
NAVIGATOR 2010	?	+	+	?	+	?
NICOLE 2003	+	+	+	+	+	+
NILVAD 2018	?	?	+	?	+	?
The Norwegian Multicenter Study 1981	+	+	+	+	+	+
ONTARGET 2008	+	+	+	+	+	+
ORIENT 2011	+	+	+	+	+	+
PEACE 2004	+	+	+	+	+	+
PRoFESS 2008	?	+	+	+	+	?
PROGRESS 2001	?	–	–	+	?	–
ROADMAP 2011/12	+	+	+	+	+	+
SANDS 2009	–	?	+	–	+	–
SENIORS 2005	?	?	+	+	+	?

SHEP 1991	+	+	+	+	+	+
SHEP 2000	?	?	+	+	+	?
SOLVD 1992	+	+	+	+	+	+
Spironolactone in mild heart failure 2016	+	+	+	+	+	+
SPRINT 2015	?	?	–	+	–	–
SPS3 2013	+	+	+	+	+	+
TRACE 1995	+	+	+	+	+	+
TRANSCEND 2008	+	+	+	+	+	+
TROPHY 2006	+	+	+	+	+	+
VA NEPHRON-D 2013	+	+	+	+	+	+
Val-HeFT 2001	+	+	+	+	+	+
VALIANT 2003	?	+	+	+	+	?
VA-NHLBI 1978	+	+	+	+	+	+

+ = Low risk

? = Some concerns

– = High risk

Supplementary figure 1. Random effects meta-analysis of randomised controlled trials examining the association between antihypertensive treatment and falls by drug class

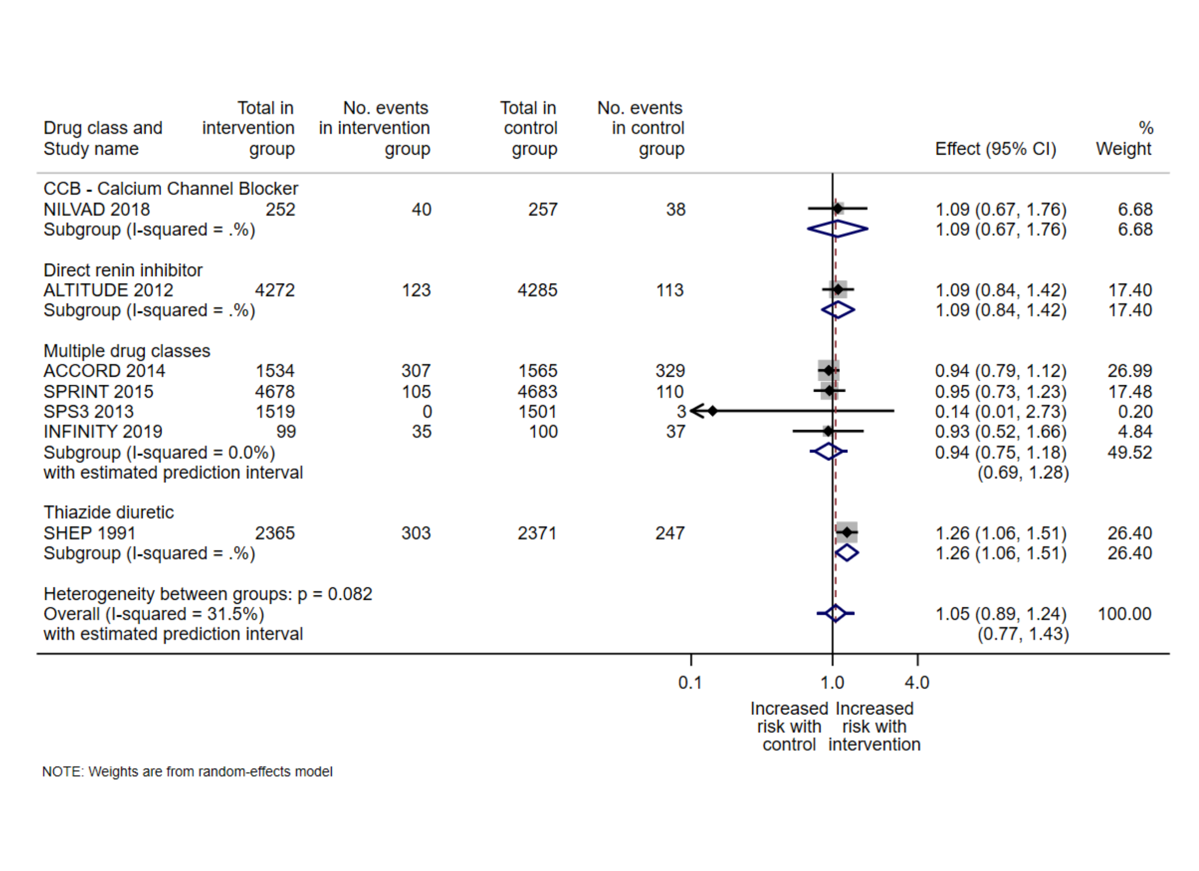

Supplementary figure 2. Random effects meta-analysis of randomised controlled trials examining the association between antihypertensive treatment and hyperkalaemia

Outcome data type and Study name	Total in intervention group	No. events in intervention group	Total in control group	No. events in control group	Effect (95% CI)	% Weight
Binary outcomes						
SOLVD 1991	1285	82	1284	32	2.67 (1.76, 4.04)	5.42
CONSENSUS II 1992	3044	12	3046	2	6.02 (1.35, 26.94)	1.28
TRACE 1995	876	43	873	23	1.91 (1.14, 3.19)	4.73
HT in Diabetes IV 1996	448	27	235	14	1.01 (0.52, 1.97)	3.81
The ACE Inhibition in PRI Study 1996	300	5	283	3	1.58 (0.37, 6.68)	1.36
IDNT 2001 Amlodipine vs placebo	565	3	284	1	1.51 (0.16, 14.59)	0.62
IDNT 2001 Irbesartan vs placebo	579	11	284	1	5.48 (0.70, 42.66)	0.74
CHARM 2003	1514	22	1509	9	2.46 (1.13, 5.35)	3.23
CHARM-Alternative 2003	1013	19	1015	3	6.45 (1.90, 21.86)	1.77
CHARM-ADDED 2003	1276	44	1272	9	5.01 (2.44, 10.31)	3.51
VALIANT 2003 Combination vs Captopril only	2431	6	4879	4	3.02 (0.85, 10.70)	1.67
VALIANT 2003 Combination vs Valsartan only	2431	6	4885	7	1.72 (0.58, 5.14)	2.09
HOPE 2007	4645	8	4652	6	1.34 (0.46, 3.85)	2.18
TRANSCEND 2008	2954	111	2972	49	2.33 (1.66, 3.27)	5.95
I-PRESERVE 2008	2067	62	2061	41	1.52 (1.02, 2.27)	5.53
PRoFRESS 2008	10146	14	10186	6	2.34 (0.90, 6.10)	2.51
SANDS 2009	276	2	272	0	5.01 (0.24, 105.72)	0.35
GISSI-AF 2009	722	40	730	18	2.32 (1.32, 4.09)	4.40
NAVIGATOR 2010	4631	35	4675	35	1.01 (0.63, 1.62)	5.04
ACCORD 2010	2362	73	2371	72	1.02 (0.73, 1.42)	6.02
ROADMAP 2011	2232	11	2215	8	1.37 (0.55, 3.40)	2.67
EMPHASIS-HF 2011	1360	109	1369	50	2.30 (1.63, 3.24)	5.93
ASPIRE 2011	422	55	397	26	2.14 (1.31, 3.48)	4.91
ORIENT 2011	282	26	284	15	1.82 (0.94, 3.52)	3.85
ALTITUDE 2012	4272	1670	4285	1244	1.57 (1.43, 1.72)	7.30
Wu 2016	69	5	70	2	2.66 (0.50, 14.18)	1.06
Subgroup (I-squared = 61.0%)					1.92 (1.59, 2.33)	87.94
with estimated prediction interval					(1.00, 3.69)	
Time-to-event outcomes						
VA NEPHRON-D 2013	724	72	724	32	2.80 (1.80, 4.30)	5.28
SPRINT 2015	4678	176	4683	171	1.00 (0.81, 1.23)	6.78
Subgroup (I-squared = 94.3%)					1.64 (0.00, 1134.06)	12.06
Heterogeneity between groups: p = 0.002						
Overall (I-squared = 71.8%)					1.89 (1.56, 2.30)	100.00
with estimated prediction interval					(0.90, 3.98)	

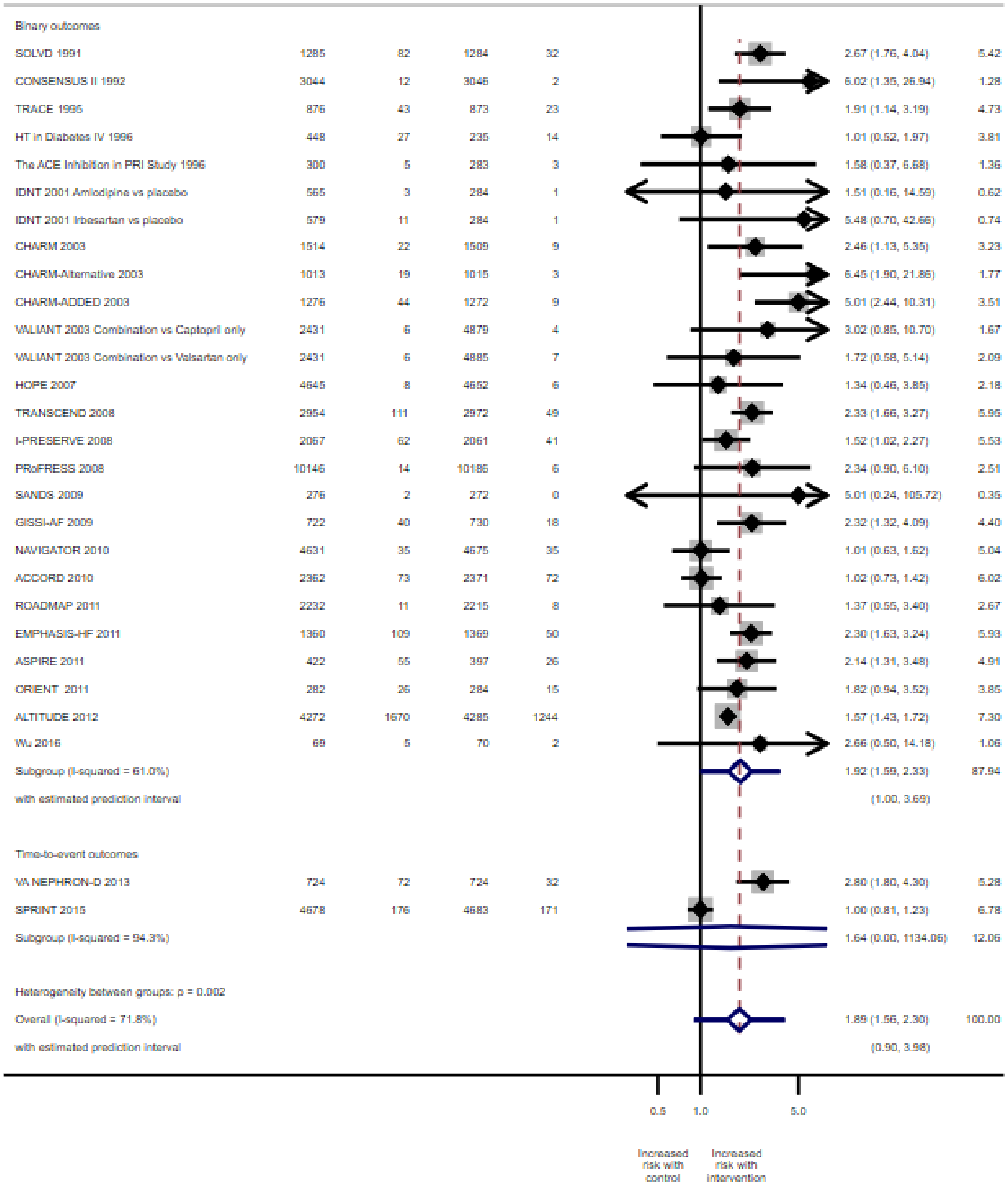

NOTE: Weights are from random-effects model

Supplementary figure 3. Random effects meta-analysis of randomised controlled trials examining the association between antihypertensive treatment and hypotension

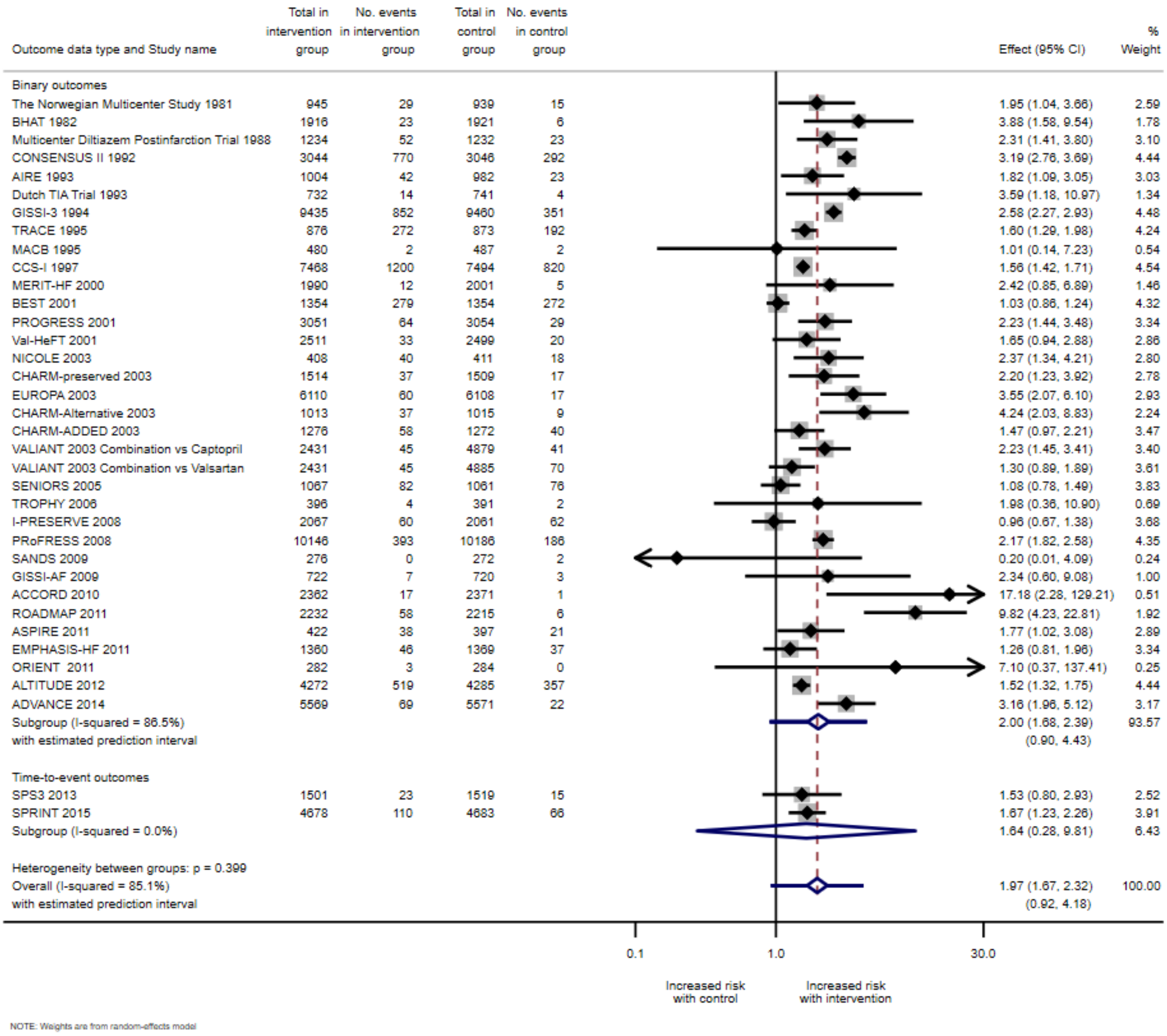

Supplementary figure 4. Random effects meta-analysis of randomised controlled trials examining the association between antihypertensive treatment and syncope

Outcome data type and Study name	Total in intervention group	No. events in intervention group	Total in control group	No. events in control group	Effect (95% CI)	% Weight
Binary outcomes						
The Norwegian Multicenter Study 1981	945	10	939	13	0.76 (0.33, 1.75)	4.61
BHAT 1982	1916	2	1921	2	1.00 (0.14, 7.12)	1.01
AIRE 1993	1004	24	982	17	1.39 (0.74, 2.60)	6.93
BEST 2001	1354	131	1354	137	0.95 (0.74, 1.22)	15.85
TROPHY 2006	396	2	391	1	1.98 (0.18, 21.92)	0.68
PRoFRESS 2008	10146	21	10186	6	3.52 (1.42, 8.72)	4.00
TRANSCEND 2008	2954	1	2972	0	3.01 (0.12, 73.61)	0.39
ONTARGET 2008	8502	29	8576	15	1.95 (1.05, 3.65)	6.98
SANDS 2009	276	2	272	1	1.98 (0.18, 21.94)	0.68
ACCORD 2010	2362	12	2371	5	2.42 (0.85, 6.87)	3.16
ALTITUDE 2012	4272	73	4285	86	0.85 (0.62, 1.16)	13.93
PEACE 2014	4158	200	4132	161	1.25 (1.01, 1.54)	17.10
HOPE-3 2016	6356	7	6349	4	1.75 (0.51, 5.98)	2.39
NILVAD 2018	252	12	257	10	1.23 (0.52, 2.91)	4.38
Subgroup (I-squared = 47.7%)					1.27 (0.97, 1.65)	82.07
with estimated prediction interval					(0.67, 2.38)	
Time-to-event outcomes						
SPS3 2013	1501	11	1519	5	2.18 (0.76, 6.27)	3.11
SPRINT 2015	4678	107	4683	80	1.33 (1.00, 1.77)	14.82
Subgroup (I-squared = 0.0%)					1.38 (0.23, 8.22)	17.93
Heterogeneity between groups: p = 0.234						
Overall (I-squared = 42.9%)					1.28 (1.03, 1.59)	100.00
with estimated prediction interval					(0.75, 2.17)	

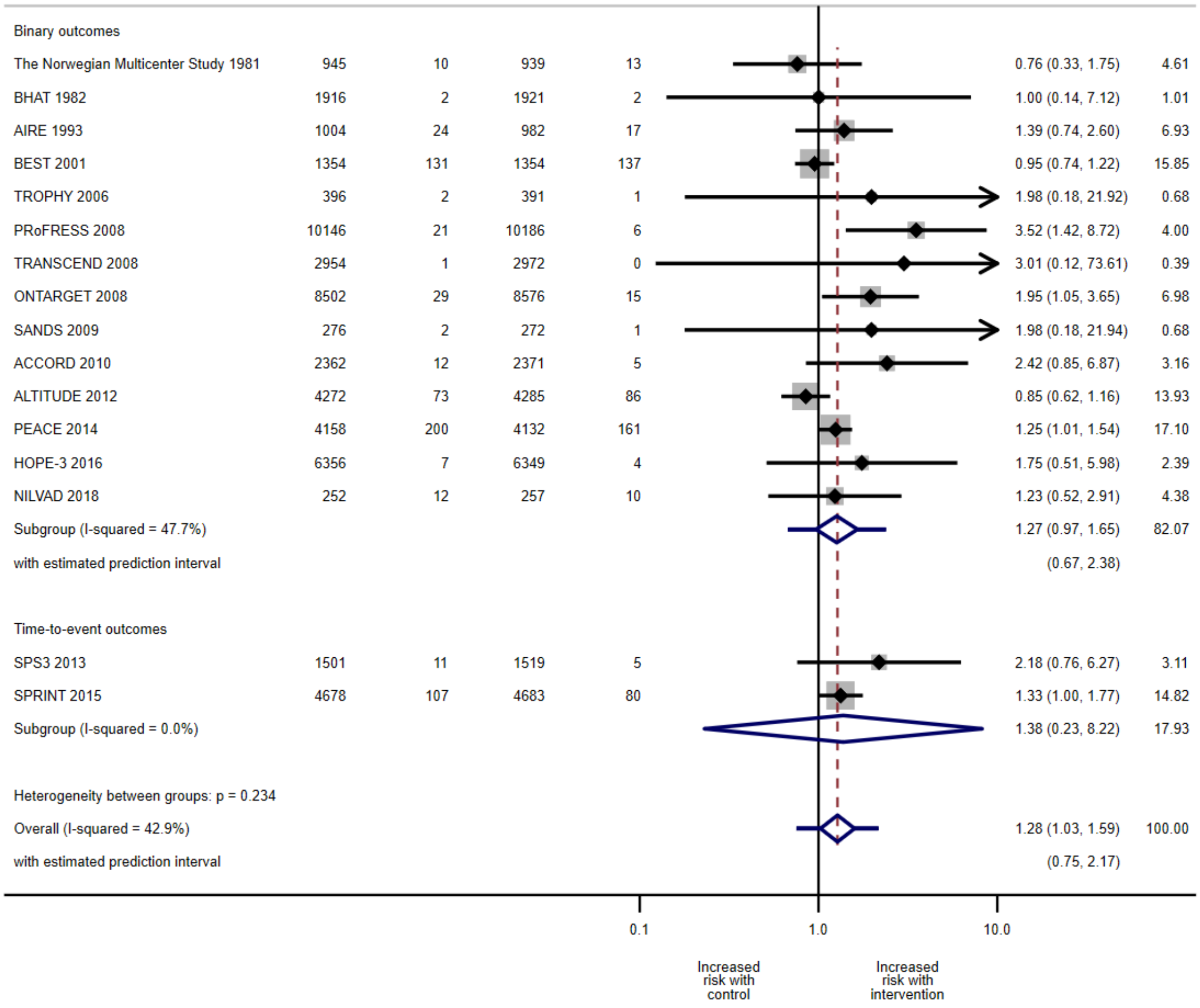

NOTE: Weights are from random-effects model

Supplementary figure 5. Random effects meta-analysis of randomised controlled trials examining the association between antihypertensive treatment and fractures

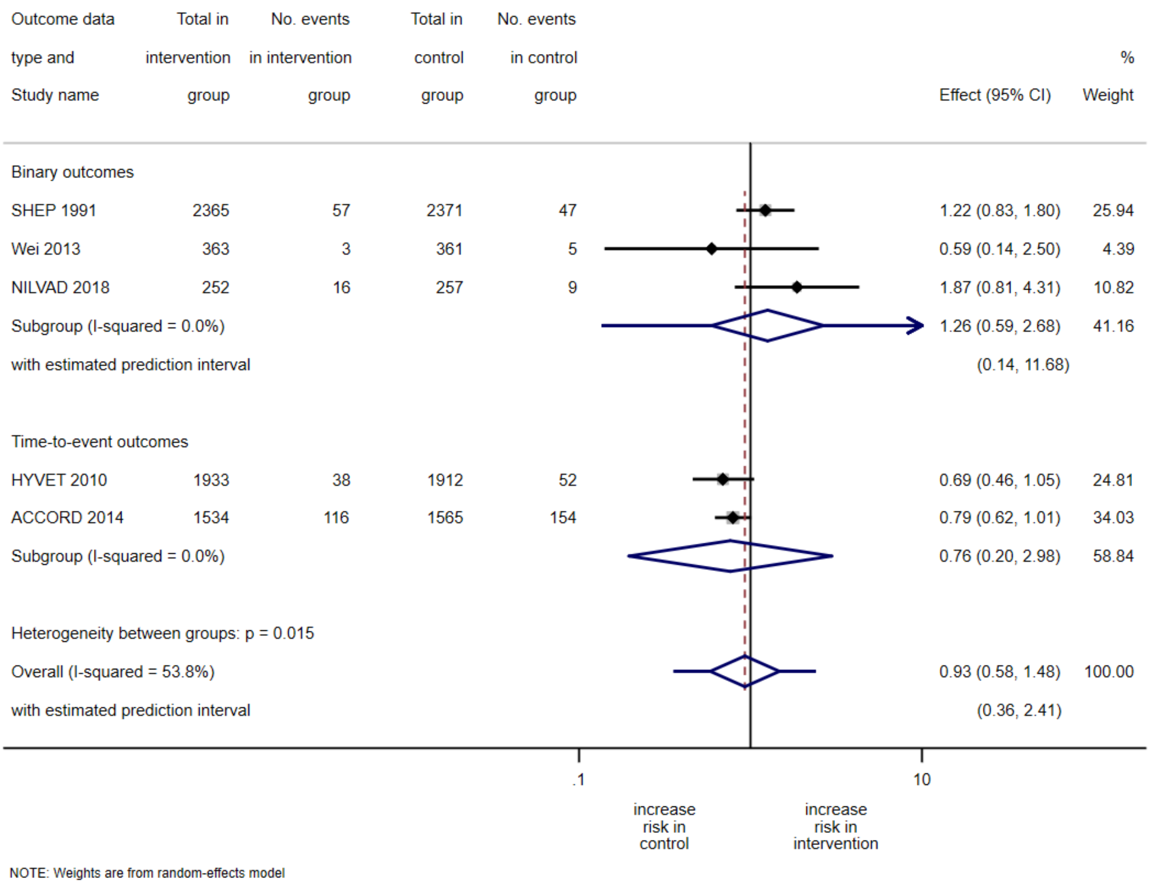

Supplementary figure 6. Random effects meta-analysis of randomised controlled trials examining the association between antihypertensive treatment and gout

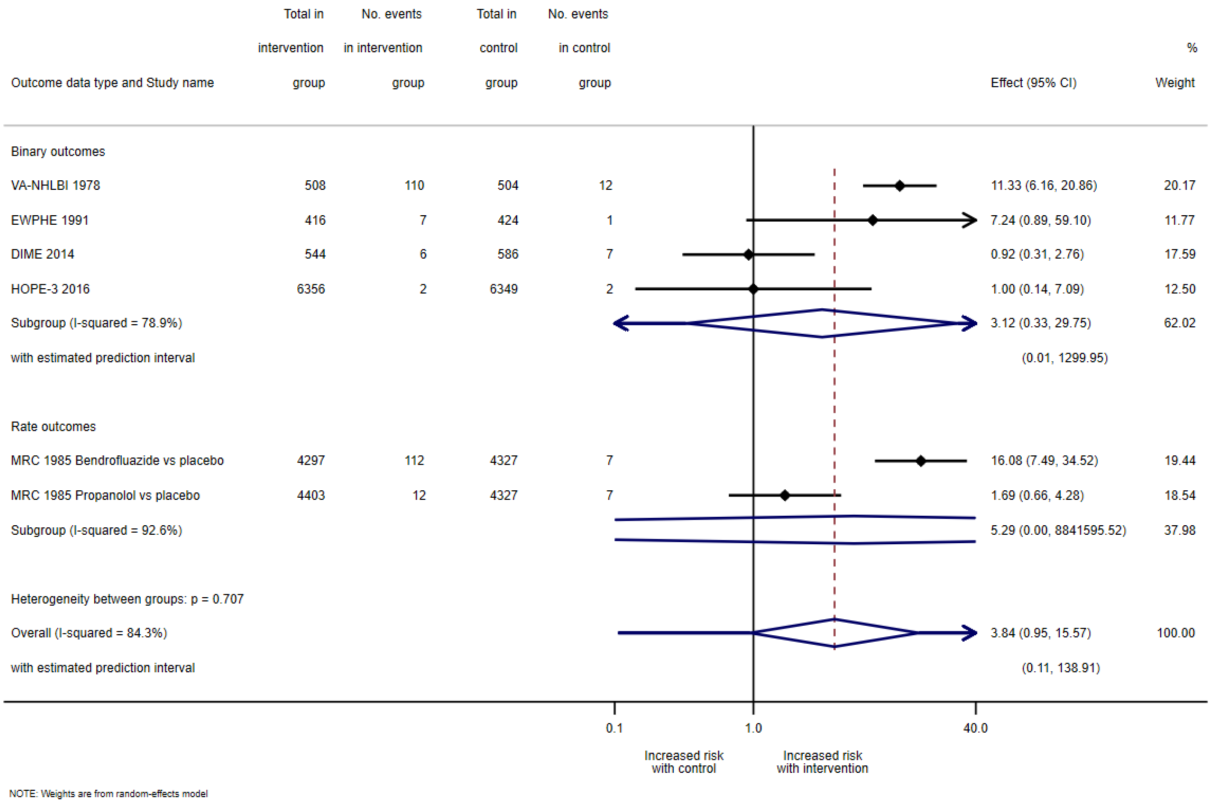

Supplementary figure 7. Random effects meta-analysis of randomised controlled trials examining the association between antihypertensive treatment and hypokalaemia

Outcome data type and Study name	Total in intervention group	No. events in intervention group	Total in control group	No. events in control group	Effect (95% CI)	% Weight
Binary outcomes						
VA-NHLBI 1978	508	106	504	3	44.03 (13.87, 139.76)	8.30
HT in Diabetes IV 1996	448	9	235	7	0.67 (0.25, 1.82)	8.68
SHEP 2000	2102	151	2024	21	7.38 (4.66, 11.70)	9.75
I-PRESERVE 2008	2067	12	2061	9	1.33 (0.56, 3.17)	8.99
Cardio-Sis 2009	557	2	553	1	1.99 (0.18, 22.00)	5.22
NAVIGATOR 2010	4631	45	4675	84	0.54 (0.37, 0.77)	9.87
ACCORD 2010	2362	49	2371	27	1.84 (1.15, 2.95)	9.73
EMPHASIS-HF 2011	1360	16	1369	30	0.53 (0.29, 0.98)	9.51
ASPIRE 2011	422	10	397	11	0.85 (0.36, 2.03)	8.99
DIME 2014	544	2	586	1	2.16 (0.20, 23.87)	5.22
Wu 2016	69	1	70	6	0.16 (0.02, 1.34)	5.79
Subgroup (I-squared = 93.3%)					1.54 (0.57, 4.20)	90.05
with estimated prediction interval					(0.06, 39.27)	
Time-to-event outcomes						
SPRINT 2015	4678	114	4683	74	1.50 (1.12, 2.00)	9.95
Subgroup (I-squared = .%)					1.50 (1.12, 2.00)	9.95
Heterogeneity between groups: p = 0.704						
Overall (I-squared = 94.3%)					1.54 (0.63, 3.75)	100.00
with estimated prediction interval					(0.08, 29.98)	

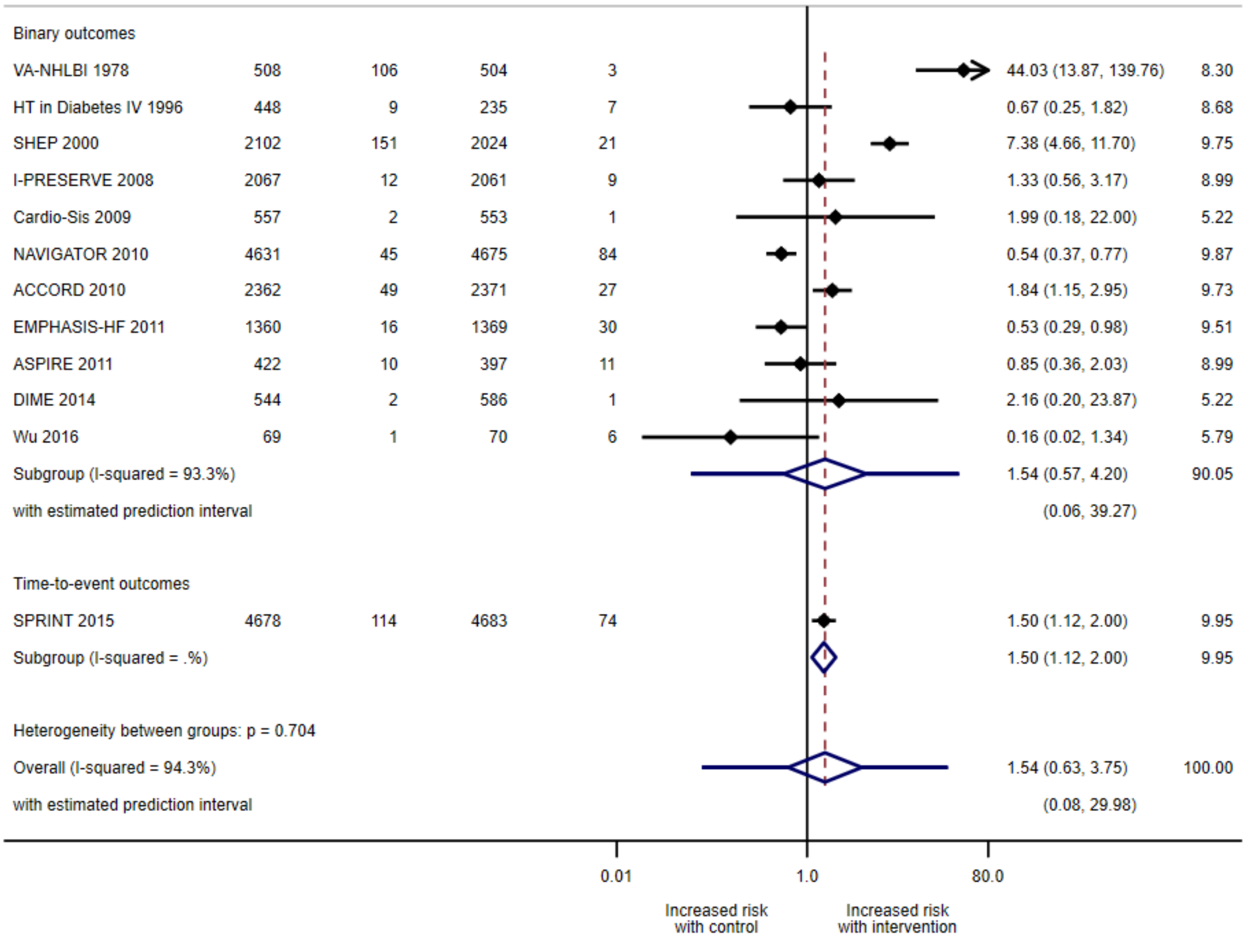

NOTE: Weights are from random-effects model

Supplementary figure 8. Random effects meta-analysis of randomised controlled trials examining the association between antihypertensive treatment and acute kidney injury by drug class

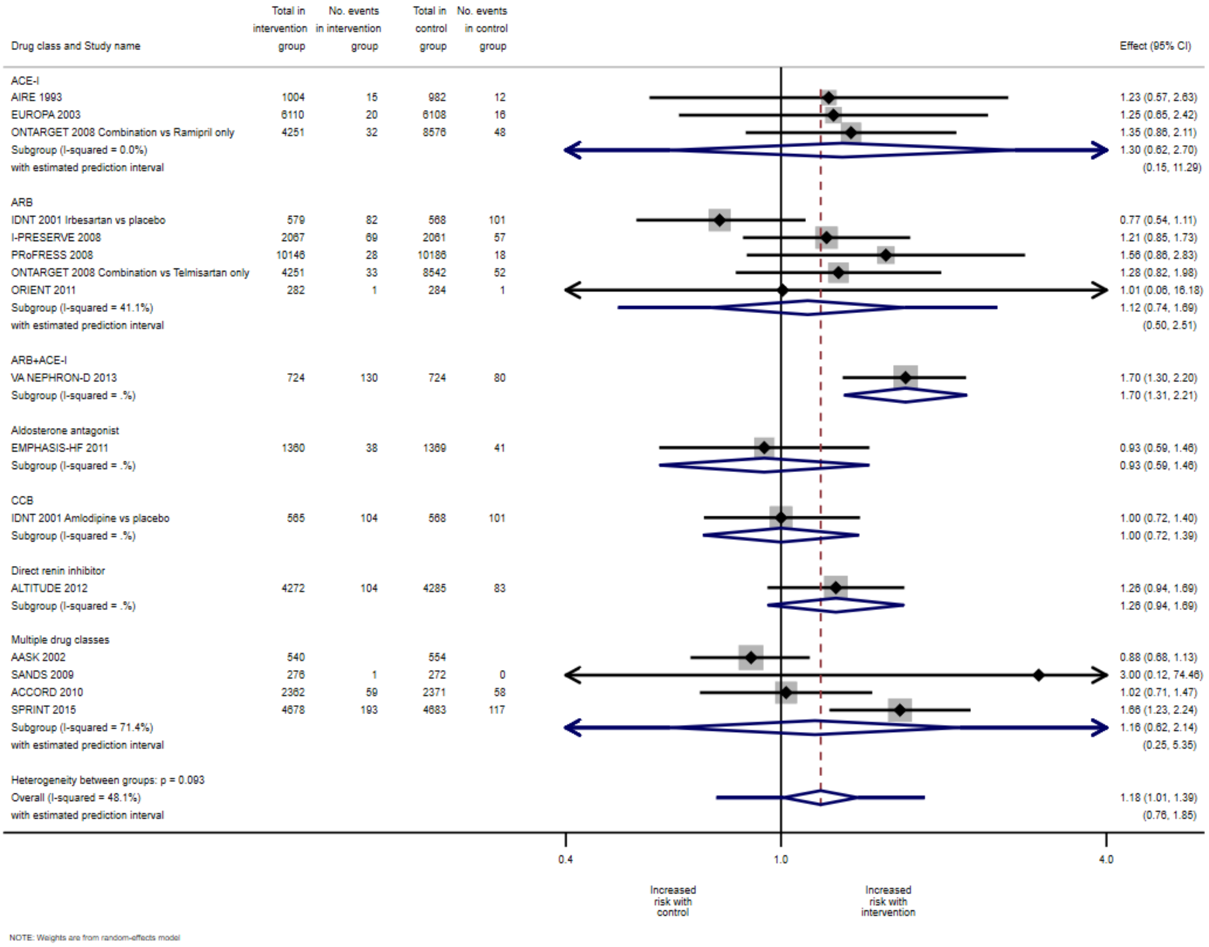

Supplementary figure 9. Random effects meta-analysis of randomised controlled trials examining the association between antihypertensive treatment and hyperkalaemia by drug class

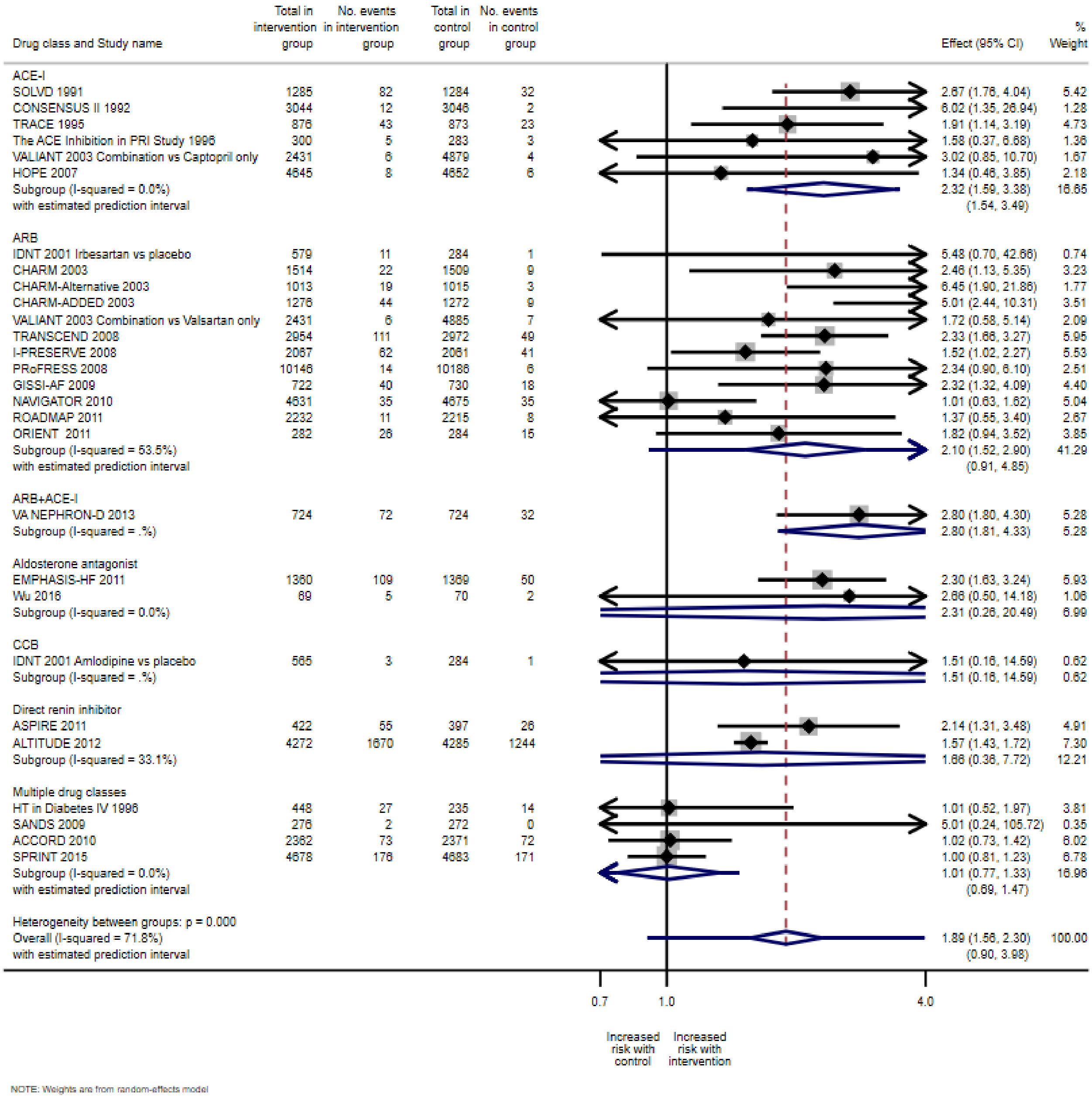

Supplementary figure 10. Random effects meta-analysis of randomised controlled trials examining the association between antihypertensive treatment and gout by drug class

Drug class and Study name	Total in intervention group	No. events in intervention group	Total in control group	No. events in control group	Effect (95% CI)	% Weight
ARB + Thiazide						
HOPE-3 2016	6356	2	6349	2	1.00 (0.14, 7.09)	12.50
Subgroup (I-squared = .%)					1.00 (0.14, 7.09)	12.50
Beta blocker						
MRC 1985 Propanolol vs placebo	4403	12	4327	7	1.69 (0.66, 4.28)	18.54
Subgroup (I-squared = .%)					1.69 (0.66, 4.28)	18.54
Thiazide diuretic						
VA-NHLBI 1978	508	110	504	12	11.33 (6.16, 20.86)	20.17
MRC 1985 Bendrofluazide vs placebo	4297	112	4327	7	16.08 (7.49, 34.52)	19.44
DIME 2014	544	6	586	7	0.92 (0.31, 2.76)	17.59
Subgroup (I-squared = 92.8%)					5.78 (0.13, 262.72)	57.20
with estimated prediction interval					(0.00, 14264143878.98)	
Thiazide diuretic + potassium sparing diuretic						
EWPHE 1991	416	7	424	1	7.24 (0.89, 59.10)	11.77
Subgroup (I-squared = .%)					7.24 (0.89, 59.10)	11.77
Heterogeneity between groups: p = 0.005						
Overall (I-squared = 84.3%)					3.84 (0.95, 15.57)	100.00
with estimated prediction interval					(0.11, 138.91)	

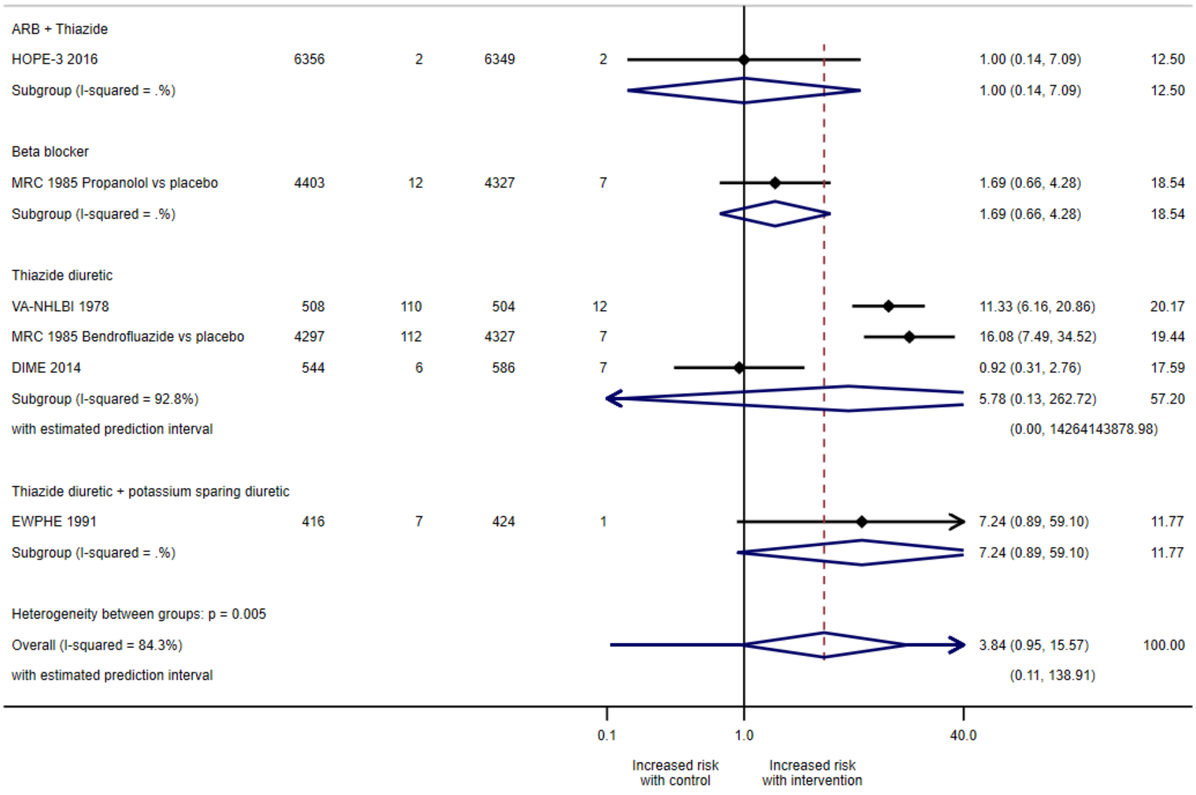

NOTE: Weights are from random-effects model

Supplementary figure 11. Random effects meta-analysis of randomised controlled trials examining the association between antihypertensive treatment and hypokalaemia by drug class

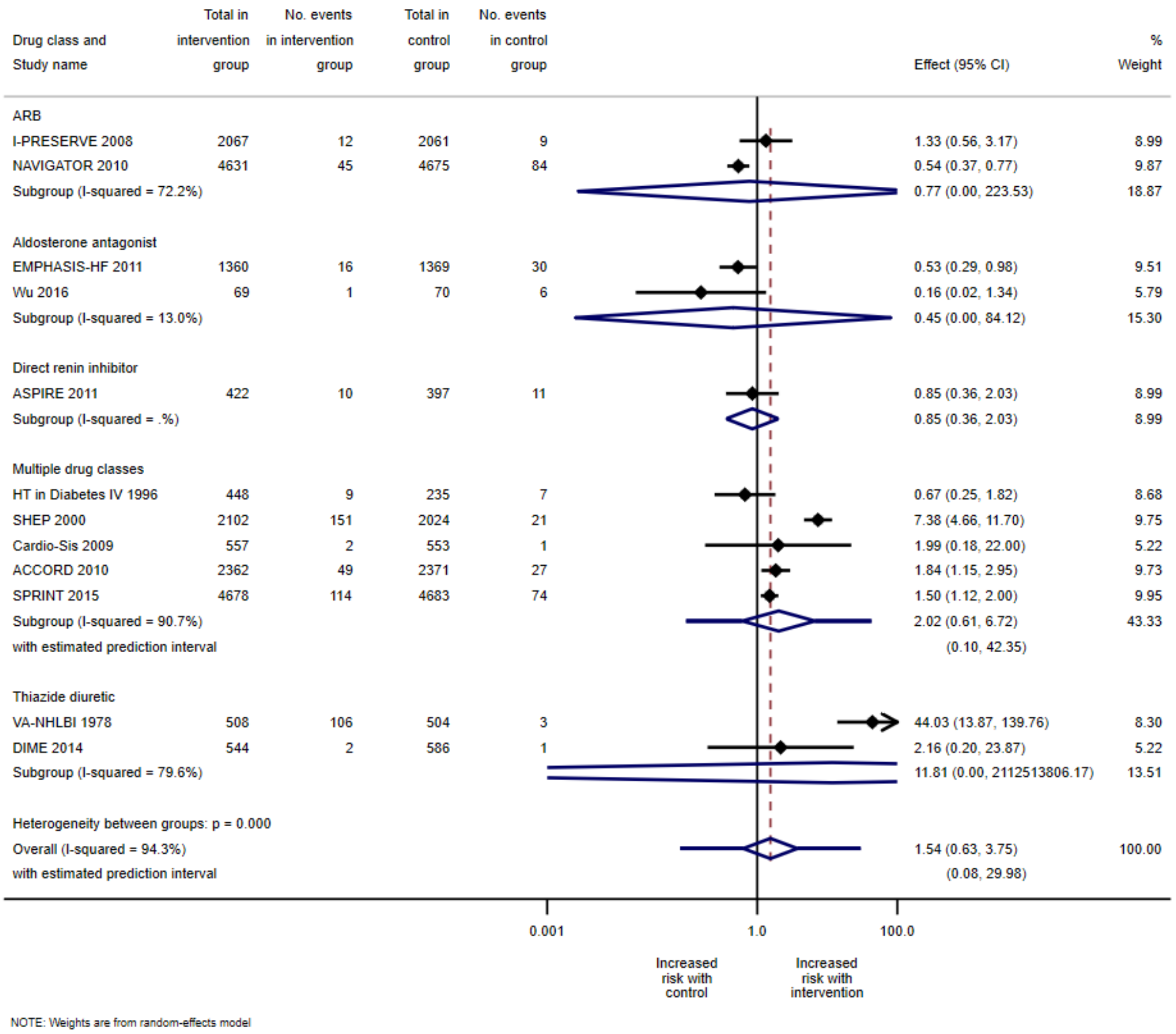

Supplementary figure 12. Random effects meta-analysis of randomised controlled trials examining the association between antihypertensive treatment and fracture by drug class

Drug class and Study name	Total in intervention group	No. events in intervention group	Total in control group	No. events in control group	Effect (95% CI)	% Weight
Thiazide diuretic ± ACE inhibitor						
HYVET 2010	1933	38	1912	52	0.69 (0.46, 1.05)	24.81
Subgroup (I-squared = .%)					0.69 (0.46, 1.04)	24.81
CCB - Calcium Channel Blocker						
NILVAD 2018	252	16	257	9	1.87 (0.81, 4.31)	10.82
Subgroup (I-squared = .%)					1.87 (0.81, 4.31)	10.82
Multiple drug classes						
Wei 2013	363	3	361	5	0.59 (0.14, 2.50)	4.39
ACCORD 2014	1534	116	1565	154	0.79 (0.62, 1.01)	34.03
Subgroup (I-squared = 0.0%)					0.78 (0.16, 3.73)	38.43
Thiazide diuretic						
SHEP 1991	2365	57	2371	47	1.22 (0.83, 1.80)	25.94
Subgroup (I-squared = .%)					1.22 (0.83, 1.80)	25.94
Heterogeneity between groups: p = 0.045						
Overall (I-squared = 53.8%)					0.93 (0.58, 1.48)	100.00
with estimated prediction interval					(0.36, 2.41)	

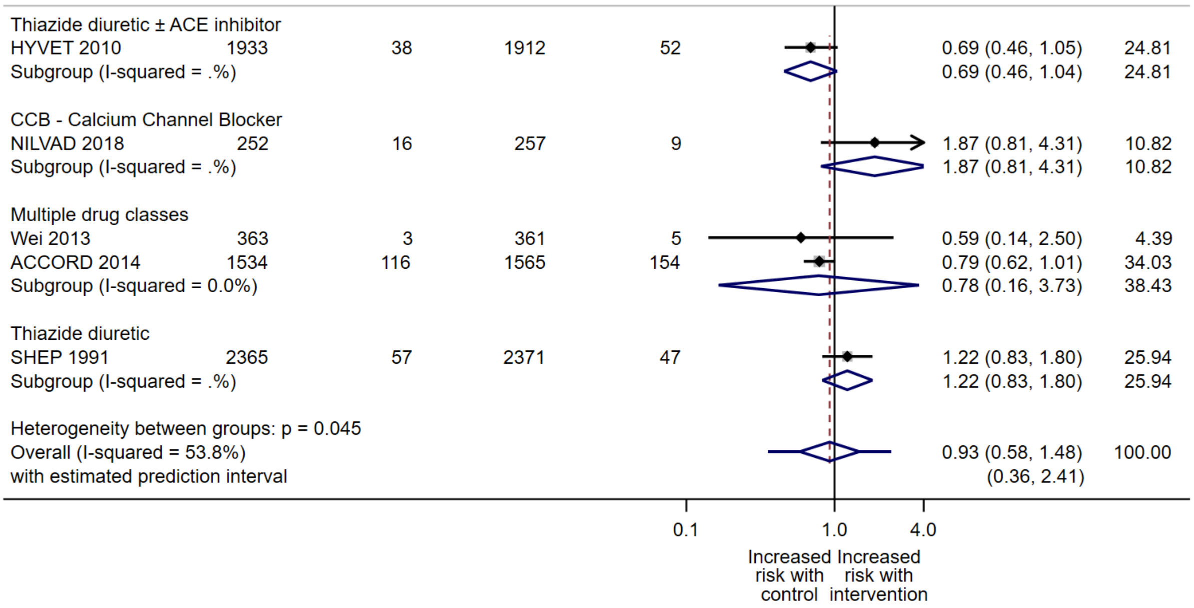

NOTE: Weights are from random-effects model

Supplementary figure 13. Random effects meta-analysis of randomised controlled trials examining the association between antihypertensive treatment and hypotension by drug class

Drug class and Study name	Total in intervention group	No. events in intervention group	Total in control group	No. events in control group	Effect (95% CI)	% Weight
ACE-I						
CONSENSUS II 1992	3044	770	3046	292	3.19 (2.76, 3.69)	4.44
AIRE 1993	1004	42	982	23	1.82 (1.09, 3.05)	3.03
GISSI-3 1994	9435	852	9460	351	2.58 (2.27, 2.93)	4.48
TRACE 1995	876	272	873	192	1.60 (1.29, 1.98)	4.24
CCS-I 1997	7468	1200	7494	820	1.56 (1.42, 1.71)	4.54
EUROPA 2003	6110	60	6108	17	3.55 (2.07, 6.10)	2.93
VALIANT 2003 Combination vs Captopril	2431	45	4879	41	2.23 (1.45, 3.41)	3.40
Subgroup (I-squared = 91.1%)					2.22 (1.62, 3.03)	27.05
with estimated prediction interval					(0.96, 5.11)	
ACE-I + Thiazide						
PROGRESS 2001	3051	64	3054	29	2.23 (1.44, 3.48)	3.34
Subgroup (I-squared = .%)					2.23 (1.44, 3.48)	3.34
ARB						
Val-HeFT 2001	2511	33	2499	20	1.65 (0.94, 2.88)	2.86
CHARM-preserved 2003	1514	37	1509	17	2.20 (1.23, 3.92)	2.78
CHARM-Alternative 2003	1013	37	1015	9	4.24 (2.03, 8.83)	2.24
CHARM-ADDED 2003	1276	58	1272	40	1.47 (0.97, 2.21)	3.47
VALIANT 2003 Combination vs Valsartan	2431	45	4885	70	1.30 (0.89, 1.89)	3.61
TROPHY 2006	396	4	391	2	1.98 (0.36, 10.90)	0.69
I-PRESERVE 2008	2067	60	2061	62	0.96 (0.67, 1.38)	3.68
PRoFRESS 2008	10146	393	10186	186	2.17 (1.82, 2.58)	4.35
GISSI-AF 2009	722	7	720	3	2.34 (0.60, 9.08)	1.00
ROADMAP 2011	2232	58	2215	6	9.82 (4.23, 22.81)	1.92
ORIENT 2011	282	3	284	0	7.10 (0.37, 137.41)	0.25
Subgroup (I-squared = 83.8%)					2.11 (1.34, 3.33)	26.84
with estimated prediction interval					(0.56, 8.01)	
Aldosterone antagonist						
EMPHASIS-HF 2011	1360	46	1369	37	1.26 (0.81, 1.96)	3.34
Subgroup (I-squared = .%)					1.26 (0.81, 1.96)	3.34
Beta blocker						
The Norwegian Multicenter Study 1981	945	29	939	15	1.95 (1.04, 3.66)	2.59
BHAT 1982	1916	23	1921	6	3.88 (1.58, 9.54)	1.78
Dutch TIA Trial 1993	732	14	741	4	3.59 (1.18, 10.97)	1.34
MACB 1995	480	2	487	2	1.01 (0.14, 7.23)	0.54
MERIT-HF 2000	1990	12	2001	5	2.42 (0.85, 6.89)	1.46
BEST 2001	1354	279	1354	272	1.03 (0.86, 1.24)	4.32
SENIORS 2005	1067	82	1061	76	1.08 (0.78, 1.49)	3.83
Subgroup (I-squared = 74.9%)					1.69 (0.97, 2.94)	15.85
with estimated prediction interval					(0.46, 6.17)	
CCB						
Multicenter Diltiazem Postinfarction Trial 1988	1234	52	1232	23	2.31 (1.41, 3.80)	3.10
NICOLE 2003	408	40	411	18	2.37 (1.34, 4.21)	2.80
Subgroup (I-squared = 0.0%)					2.34 (0.20, 26.75)	5.90
CCB+ACE-I+Thiazide						
ADVANCE 2014	5569	69	5571	22	3.16 (1.96, 5.12)	3.17
Subgroup (I-squared = .%)					3.16 (1.96, 5.12)	3.17
Direct renin inhibitor						
ASPIRE 2011	422	38	397	21	1.77 (1.02, 3.08)	2.89
ALTITUDE 2012	4272	519	4285	357	1.52 (1.32, 1.75)	4.44
Subgroup (I-squared = 0.0%)					1.54 (0.63, 3.75)	7.33
Multiple drug classes						
SANDS 2009	276	0	272	2	0.20 (0.01, 4.09)	0.24
ACCORD 2010	2362	17	2371	1	17.18 (2.28, 129.21)	0.51
SPS3 2013	1501	23	1519	15	1.53 (0.80, 2.93)	2.52
SPRINT 2015	4678	110	4683	66	1.67 (1.23, 2.26)	3.91
Subgroup (I-squared = 0.0%)					1.69 (0.86, 3.33)	7.18
with estimated prediction interval					(0.67, 4.22)	
Heterogeneity between groups: p = 0.000						
Overall (I-squared = 85.1%)					1.97 (1.67, 2.32)	100.00
with estimated prediction interval					(0.92, 4.18)	

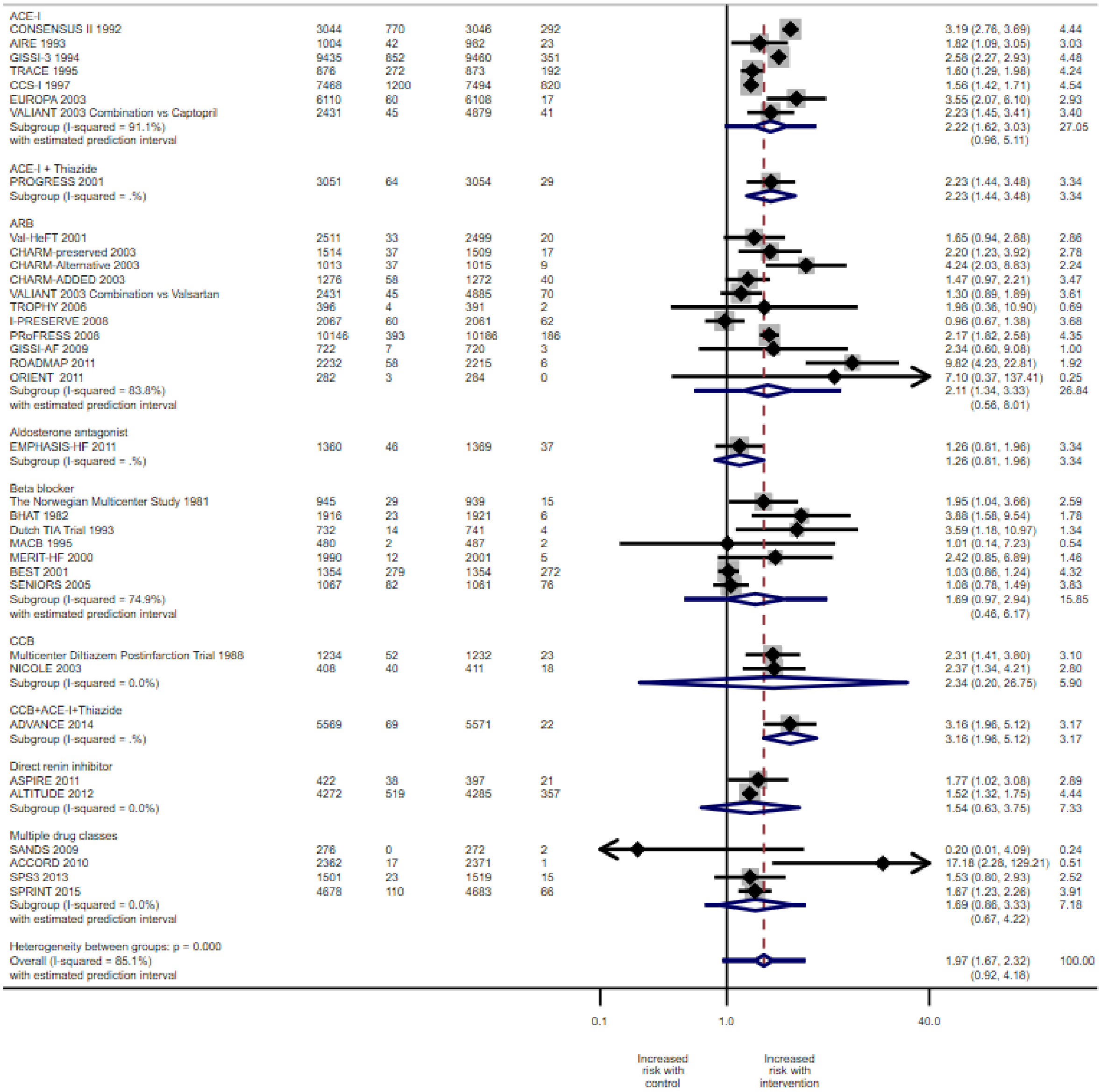

NOTE: Weights are from random-effects model

Supplementary figure 14. Random effects meta-analysis of randomised controlled trials examining the association between antihypertensive treatment and syncope by drug class

Drug class and Study name	Total in intervention group	No. events in intervention group	Total in control group	No. events in control group	Effect (95% CI)	% Weight
ACE-I						
AIRE 1993	1004	24	982	17	1.39 (0.74, 2.60)	6.93
ONTARGET 2008	8502	29	8576	15	1.95 (1.05, 3.65)	6.98
PEACE 2014	4158	200	4132	161	1.25 (1.01, 1.54)	17.10
Subgroup (I-squared = 3.9%)					1.32 (0.84, 2.08)	31.00
with estimated prediction interval					(0.30, 5.78)	
ARB						
TROPHY 2006	396	2	391	1	1.98 (0.18, 21.92)	0.68
PRoFRESS 2008	10146	21	10186	6	3.52 (1.42, 8.72)	4.00
TRANSCEND 2008	2954	1	2972	0	3.01 (0.12, 73.61)	0.39
Subgroup (I-squared = 0.0%)					3.26 (0.54, 19.74)	5.07
with estimated prediction interval					(0.02, 666.27)	
ARB + Thiazide						
HOPE-3 2016	6356	7	6349	4	1.75 (0.51, 5.98)	2.39
Subgroup (I-squared = .%)					1.75 (0.51, 5.98)	2.39
Beta blocker						
The Norwegian Multicenter Study 1981	945	10	939	13	0.76 (0.33, 1.75)	4.61
BHAT 1982	1916	2	1921	2	1.00 (0.14, 7.12)	1.01
BEST 2001	1354	131	1354	137	0.95 (0.74, 1.22)	15.85
Subgroup (I-squared = 0.0%)					0.93 (0.55, 1.58)	21.47
with estimated prediction interval					(0.20, 4.42)	
CCB						
NILVAD 2018	252	12	257	10	1.23 (0.52, 2.91)	4.38
Subgroup (I-squared = .%)					1.23 (0.52, 2.91)	4.38
Direct renin inhibitor						
ALTITUDE 2012	4272	73	4285	86	0.85 (0.62, 1.16)	13.93
Subgroup (I-squared = .%)					0.85 (0.62, 1.16)	13.93
Multiple drug classes						
SANDS 2009	276	2	272	1	1.98 (0.18, 21.94)	0.68
ACCORD 2010	2362	12	2371	5	2.42 (0.85, 6.87)	3.16
SPS3 2013	1501	11	1519	5	2.18 (0.76, 6.27)	3.11
SPRINT 2015	4678	107	4683	80	1.33 (1.00, 1.77)	14.82
Subgroup (I-squared = 10.3%)					1.52 (0.84, 2.75)	21.77
with estimated prediction interval					(0.53, 4.35)	
Heterogeneity between groups: p = 0.008						
Overall (I-squared = 42.9%)					1.28 (1.03, 1.59)	100.00
with estimated prediction interval					(0.75, 2.17)	

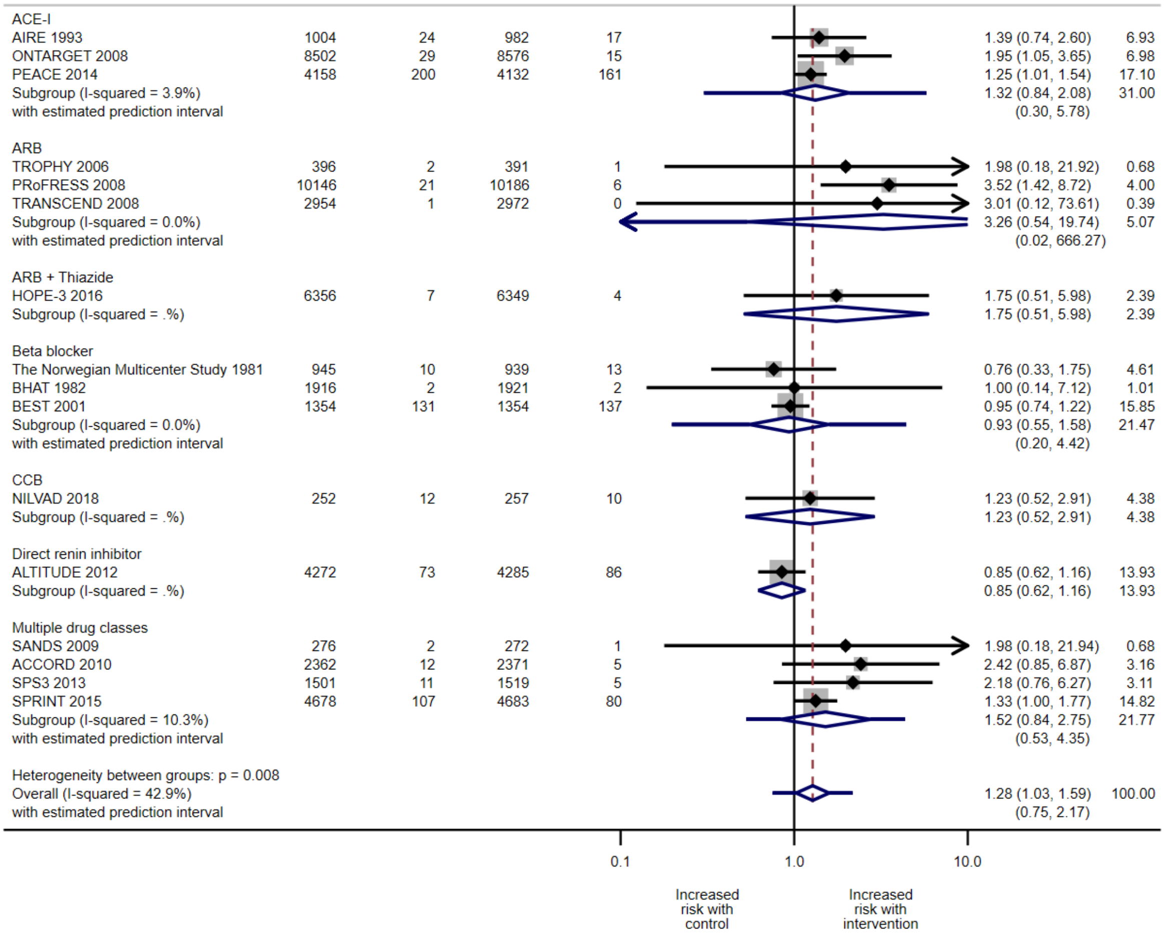

NOTE: Weights are from random-effects model

Supplementary figure 15. Random effects meta-analysis of randomised controlled trials examining the association between antihypertensive treatment and all-cause mortality

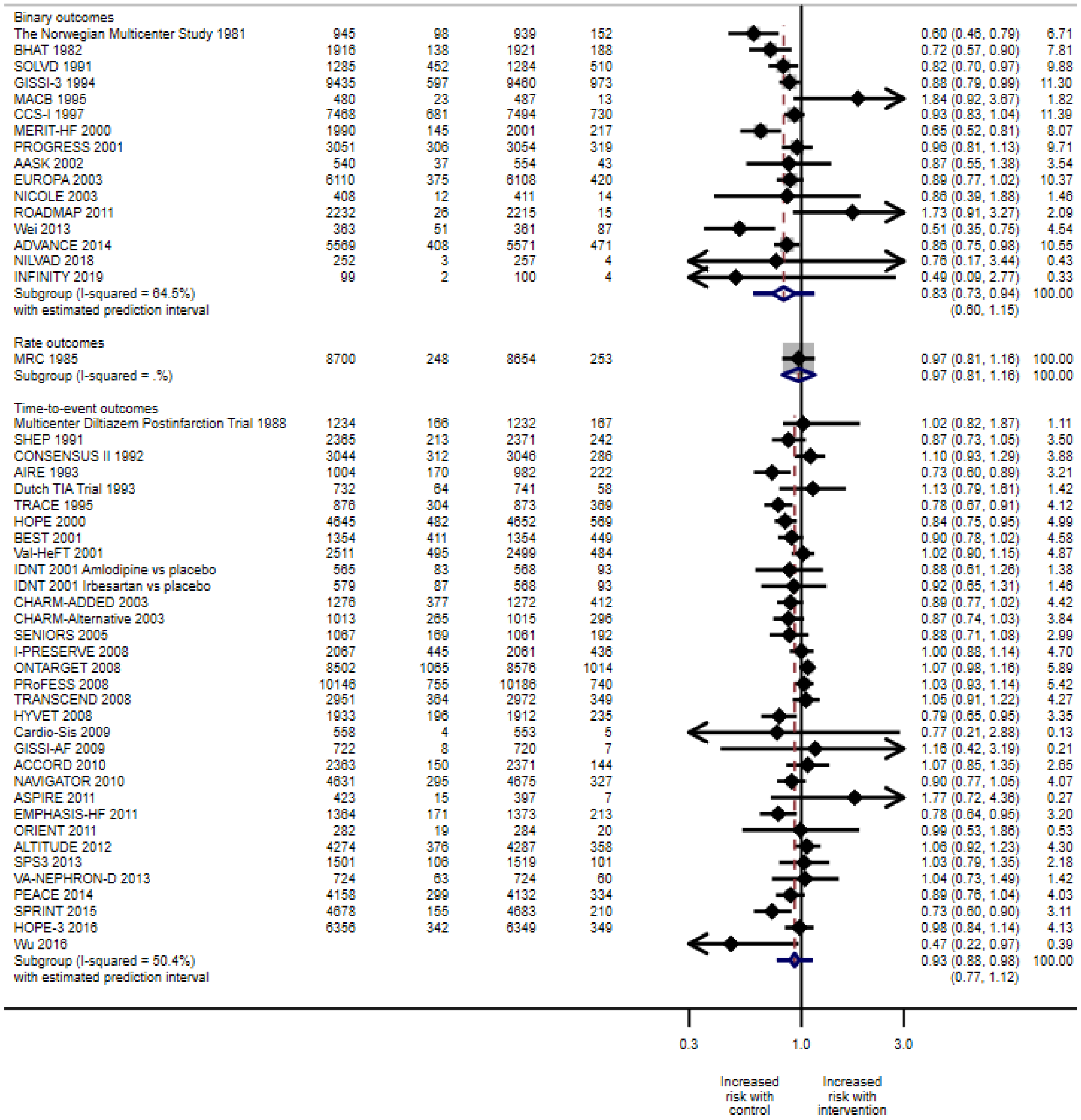

Outcome data type and Study name	Total in intervention group	No. events in intervention group	Total in control group	No. events in control group	Effect (95% CI)	% Weight
Binary outcomes						
The Norwegian Multicenter Study 1981	945	98	939	152	0.60 (0.46, 0.79)	6.71
BHAT 1982	1916	138	1921	188	0.72 (0.57, 0.90)	7.81
SOLVD 1991	1285	452	1284	510	0.82 (0.70, 0.97)	9.88
GISSI-3 1994	9435	597	9460	973	0.88 (0.79, 0.99)	11.30
MACB 1995	480	23	487	13	1.84 (0.92, 3.67)	1.82
CCS-I 1997	7468	681	7494	730	0.93 (0.83, 1.04)	11.39
MERIT-HF 2000	1990	145	2001	217	0.65 (0.52, 0.81)	8.07
PROGRESS 2001	3051	306	3054	319	0.96 (0.81, 1.13)	9.71
AASK 2002	540	37	554	43	0.87 (0.55, 1.38)	3.54
EUROPA 2003	6110	375	6108	420	0.89 (0.77, 1.02)	10.37
NICOLE 2003	408	12	411	14	0.86 (0.39, 1.88)	1.46
ROADMAP 2011	2232	26	2215	15	1.73 (0.91, 3.27)	2.09
Wei 2013	363	51	361	87	0.51 (0.35, 0.75)	4.54
ADVANCE 2014	5569	408	5571	471	0.86 (0.75, 0.98)	10.55
NILVAD 2018	252	3	257	4	0.76 (0.17, 3.44)	0.43
INFINITY 2019	99	2	100	4	0.49 (0.09, 2.77)	0.33
Subgroup (I-squared = 64.5%)					0.83 (0.73, 0.94)	100.00
with estimated prediction interval					(0.60, 1.15)	
Rate outcomes						
MRC 1985	8700	248	8654	253	0.97 (0.81, 1.16)	100.00
Subgroup (I-squared = .%)					0.97 (0.81, 1.16)	100.00
Time-to-event outcomes						
Multicenter Diltiazem Postinfarction Trial 1988	1234	166	1232	167	1.02 (0.82, 1.87)	1.11
SHEP 1991	2365	213	2371	242	0.87 (0.73, 1.05)	3.50
CONSENSUS II 1992	3044	312	3046	286	1.10 (0.93, 1.29)	3.88
AIRE 1993	1004	170	982	222	0.73 (0.60, 0.89)	3.21
Dutch TIA Trial 1993	732	64	741	58	1.13 (0.79, 1.61)	1.42
TRACE 1995	876	304	873	369	0.78 (0.67, 0.91)	4.12
HOPE 2000	4645	482	4652	569	0.84 (0.75, 0.95)	4.99
BEST 2001	1354	411	1354	449	0.90 (0.78, 1.02)	4.58
Val-HeFT 2001	2511	495	2499	484	1.02 (0.90, 1.15)	4.87
IDNT 2001 Amlodipine vs placebo	565	83	568	93	0.88 (0.61, 1.26)	1.38
IDNT 2001 Irbesartan vs placebo	579	87	568	93	0.92 (0.65, 1.31)	1.46
CHARM-ADDED 2003	1276	377	1272	412	0.89 (0.77, 1.02)	4.42
CHARM-Alternative 2003	1013	265	1015	296	0.87 (0.74, 1.03)	3.84
SENIORS 2005	1067	169	1061	192	0.88 (0.71, 1.08)	2.99
I-PRESERVE 2008	2067	445	2061	436	1.00 (0.88, 1.14)	4.70
ONTARGET 2008	8502	1065	8576	1014	1.07 (0.98, 1.16)	5.89
PRoFESS 2008	10146	755	10186	740	1.03 (0.93, 1.14)	5.42
TRANSCEND 2008	2951	364	2972	349	1.05 (0.91, 1.22)	4.27
HYVET 2008	1933	196	1912	235	0.79 (0.65, 0.95)	3.35
Cardio-Sis 2009	558	4	553	5	0.77 (0.21, 2.88)	0.13
GISSI-AF 2009	722	8	720	7	1.16 (0.42, 3.19)	0.21
ACCORD 2010	2363	150	2371	144	1.07 (0.85, 1.35)	2.65
NAVIGATOR 2010	4631	295	4675	327	0.90 (0.77, 1.05)	4.07
ASPIRE 2011	423	15	397	7	1.77 (0.72, 4.36)	0.27
EMPHASIS-HF 2011	1364	171	1373	213	0.78 (0.64, 0.95)	3.20
ORIENT 2011	282	19	284	20	0.99 (0.53, 1.86)	0.53
ALTITUDE 2012	4274	376	4287	358	1.06 (0.92, 1.23)	4.30
SPS3 2013	1501	106	1519	101	1.03 (0.79, 1.35)	2.18
VA-NEPHRON-D 2013	724	63	724	60	1.04 (0.73, 1.49)	1.42
PEACE 2014	4158	299	4132	334	0.89 (0.76, 1.04)	4.03
SPRINT 2015	4678	155	4683	210	0.73 (0.60, 0.90)	3.11
HOPE-3 2016	6356	342	6349	349	0.98 (0.84, 1.14)	4.13
Wu 2016					0.47 (0.22, 0.97)	0.39
Subgroup (I-squared = 50.4%)					0.93 (0.88, 0.98)	100.00
with estimated prediction interval					(0.77, 1.12)	

NOTE: Weights are from random-effects model

Supplementary figure 16. Random effects meta-analysis of randomised controlled trials examining the association between antihypertensive treatment and stroke

Outcome data type and Study name	Total in intervention group	No. events in intervention group	Total in control group	No. events in control group	Effect (95% CI)	% Weight
Binary outcomes						
SOLVD 1991	1285	10	1284	11	0.91 (0.38, 2.14)	3.06
SHEP 1991	2371	106	2365	163	0.63 (0.49, 0.81)	12.45
AIRE 1993	1004	25	982	17	1.45 (0.78, 2.70)	5.04
GISSI-3 1994	9435	72	9460	68	1.06 (0.76, 1.48)	10.23
MACB 1995	480	4	487	5	0.81 (0.22, 3.04)	1.44
The ACE Inhibition in PRI Study 1996	300	2	283	3	0.63 (0.10, 3.78)	0.81
CCS-I 1997	7468	92	7494	90	1.03 (0.77, 1.37)	11.31
PROGRESS 2001	3051	307	3054	420	0.70 (0.60, 0.82)	15.10
CHARM-Alternative 2003	1013	36	1015	42	0.85 (0.54, 1.34)	7.56
EUROPA 2003	6110	98	6108	102	0.96 (0.73, 1.27)	11.66
CHARM-ADDED 2003	1276	47	1272	41	1.15 (0.75, 1.76)	8.10
I-PRESERVE 2008	2067	79	2061	68	1.16 (0.84, 1.62)	10.31
GISSI-AF 2009	722	4	720	0	9.04 (0.48, 168.50)	0.32
DIME 2014	544	11	586	5	2.40 (0.83, 6.95)	2.13
INFINITY 2019	99	1	100	2	0.50 (0.04, 5.60)	0.46
Subgroup (I-squared = 51.5%)					0.94 (0.79, 1.13)	100.00
with estimated prediction interval					(0.59, 1.51)	
Rate outcomes						
MRC 1985	8700	60	8654	109	0.55 (0.40, 0.75)	74.47
Wei 2013	363	21	361	36	0.53 (0.31, 0.91)	25.53
Subgroup (I-squared = 0.0%)					0.54 (0.09, 3.18)	100.00
Time-to-event outcomes						
Dutch TIA Trial 1993	732	52	741	62	0.84 (0.58, 1.22)	4.43
HOPE 2000	4645	156	4652	226	0.68 (0.56, 0.84)	9.11
ONTARGET 2008	8502	373	8576	405	0.93 (0.81, 1.07)	11.93
PRoFESS 2008	10146	880	10186	934	0.95 (0.86, 1.04)	14.00
TRANSCEND 2008	2951	112	2972	136	0.83 (0.64, 1.06)	7.32
HYVET 2008	1933	51	1912	69	0.70 (0.49, 1.01)	4.61
Cardio-Sis 2009	558	4	553	9	0.44 (0.13, 1.42)	0.57
ACCORD 2010	2363	36	2371	62	0.59 (0.39, 0.89)	3.79
NAVIGATOR 2010	4631	105	4675	132	0.79 (0.61, 1.02)	7.16
EMPHASIS-HF 2011	1364	21	1373	26	0.78 (0.44, 1.39)	2.19
ORIENT 2011	282	8	284	11	0.73 (0.29, 1.83)	0.93
ALTITUDE 2012	4274	147	4287	122	1.22 (0.96, 1.55)	7.74
SPS3 2013	1501	125	1519	152	0.81 (0.64, 1.03)	7.80
VA NEPHRON-D 2013	724	18	724	18	0.98 (0.52, 1.85)	1.85
PEACE 2014	4158	71	4132	92	0.76 (0.56, 1.04)	5.71
SPRINT 2015	4678	62	4683	70	0.89 (0.63, 1.25)	4.98
HOPE-3 2016	6356	75	6349	94	0.80 (0.59, 1.08)	5.89
Subgroup (I-squared = 44.8%)					0.84 (0.76, 0.93)	100.00
with estimated prediction interval					(0.64, 1.09)	

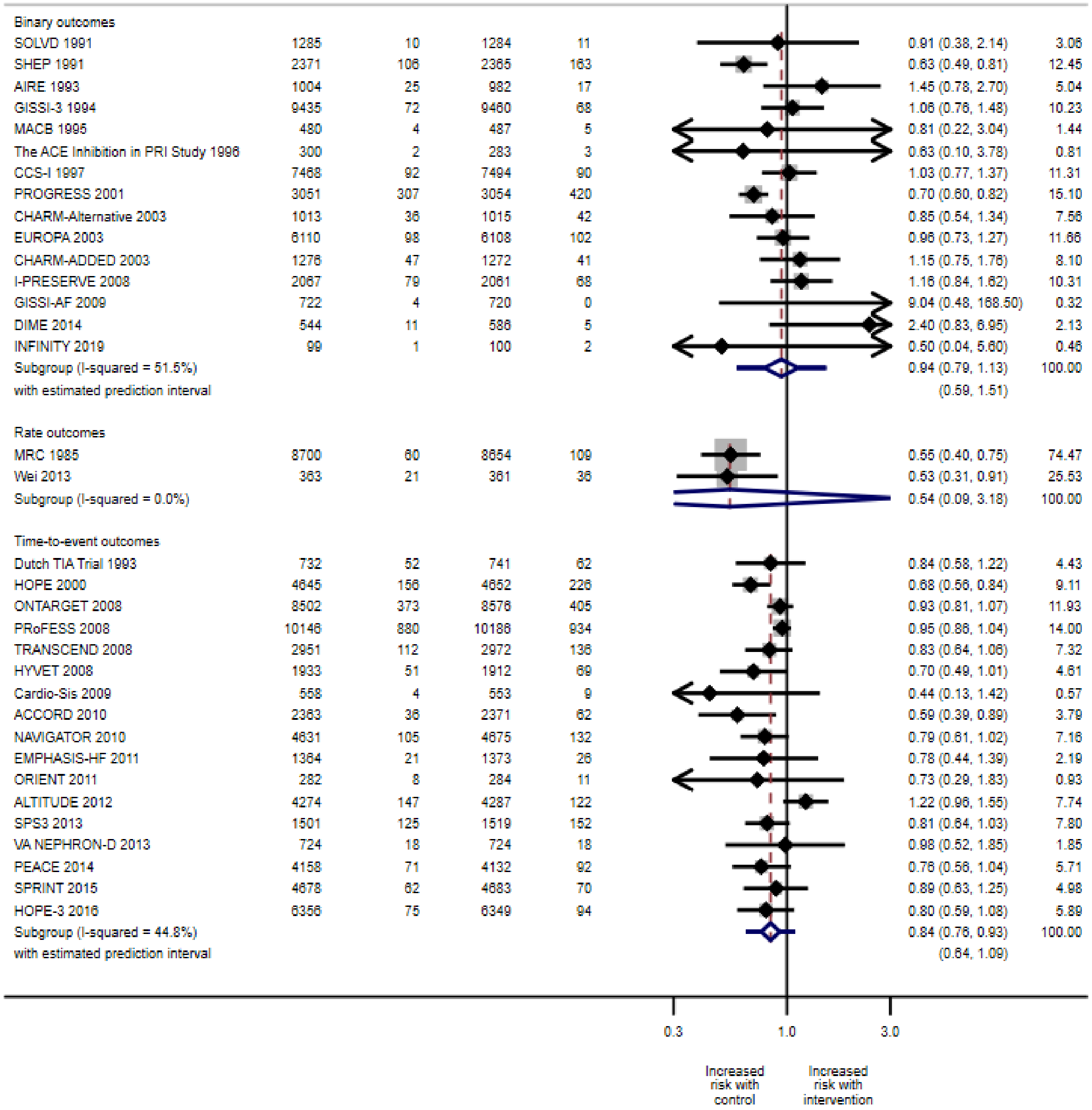

NOTE: Weights are from random-effects model

Supplementary figure 17. Random effects meta-analysis of randomised controlled trials examining the association between antihypertensive treatment and myocardial infarction

Outcome data type and Study name	Total in intervention group	No. events in intervention group	Total in control group	No. events in control group	Effect (95% CI)	% Weight
Binary outcomes						
VA-NHLBI 1978	508	6	504	5	1.19 (0.36, 3.93)	0.70
BHAT 1982	1916	8	1921	8	1.00 (0.38, 2.68)	1.04
Multicenter Diltiazem Postinfarction Trial 1988	1234	99	1232	116	0.84 (0.63, 1.11)	12.73
SOLVD 1991	1285	40	1284	53	0.75 (0.49, 1.13)	5.74
MACB 1995	480	9	487	4	2.31 (0.71, 7.54)	0.71
TRACE 1995	876	34	873	47	0.71 (0.45, 1.11)	4.91
The ACE Inhibition in PRI Study 1996	300	5	283	2	2.38 (0.46, 12.37)	0.37
MERIT-HF 2000	1990	11	2001	15	0.74 (0.34, 1.61)	1.65
PROGRESS 2001	3051	60	3054	96	0.62 (0.45, 0.86)	9.40
EUROPA 2003	6110	320	6108	418	0.75 (0.65, 0.87)	44.48
NICOLE 2003	408	16	411	13	1.25 (0.59, 2.63)	1.81
CHARM-ADDED 2003	1276	44	1272	69	0.62 (0.42, 0.92)	6.71
I-PRESERVE 2008	2067	60	2061	54	1.11 (0.77, 1.61)	7.21
Wei 2013	363	17	361	16	1.06 (0.53, 2.13)	2.05
DIME 2014	544	2	586	5	0.43 (0.08, 2.22)	0.37
INFINITY 2019	99	0	100	4	0.11 (0.01, 2.03)	0.12
Subgroup (I-squared = 0.0%)					0.78 (0.69, 0.88)	100.00
with estimated prediction interval					(0.69, 0.88)	
Time-to-event outcomes						
SHEP 1991	2365	50	2371	74	0.75 (0.54, 1.04)	4.87
TRACE 1995	876	99	873	113	0.86 (0.66, 1.13)	6.29
HOPE 2000	4645	459	4652	570	0.80 (0.70, 0.90)	11.87
BEST 2001	1354	10	1354	13	0.75 (0.33, 1.72)	1.04
CHARM-Alternative 2003	1013	75	1015	48	1.52 (1.06, 2.18)	4.25
ONTARGET 2008	8502	438	8576	413	1.08 (0.94, 1.23)	11.46
TRANSCEND 2008	2951	116	2972	147	0.79 (0.62, 1.01)	7.04
HYVET 2008	1933	9	1912	12	0.72 (0.30, 1.70)	0.95
Cardio-Sis 2009	558	4	553	6	0.66 (0.19, 2.34)	0.47
ACCORD 2010	2363	126	2371	146	0.87 (0.68, 1.10)	7.15
NAVIGATOR 2010	4631	138	4675	140	0.97 (0.77, 1.23)	7.36
EMPHASIS-HF 2011	1364	45	1373	33	1.34 (0.86, 2.10)	3.06
ORIENT 2011	282	3	284	7	0.45 (0.11, 1.75)	0.39
ALTITUDE 2012	4274	147	4287	142	1.04 (0.83, 1.31)	7.57
SPS3 2013	1501	36	1519	40	0.88 (0.56, 1.39)	2.97
VA NEPHRON-D 2013	724	52	724	40	1.30 (0.87, 1.97)	3.52
PEACE 2014	4158	222	4132	220	1.00 (0.83, 1.20)	9.24
SPRINT 2015	4678	97	4683	116	0.83 (0.64, 1.09)	6.37
HOPE-3 2016	6356	52	6349	62	0.84 (0.58, 1.21)	4.13
Subgroup (I-squared = 40.7%)					0.94 (0.85, 1.03)	100.00
with estimated prediction interval					(0.73, 1.21)	

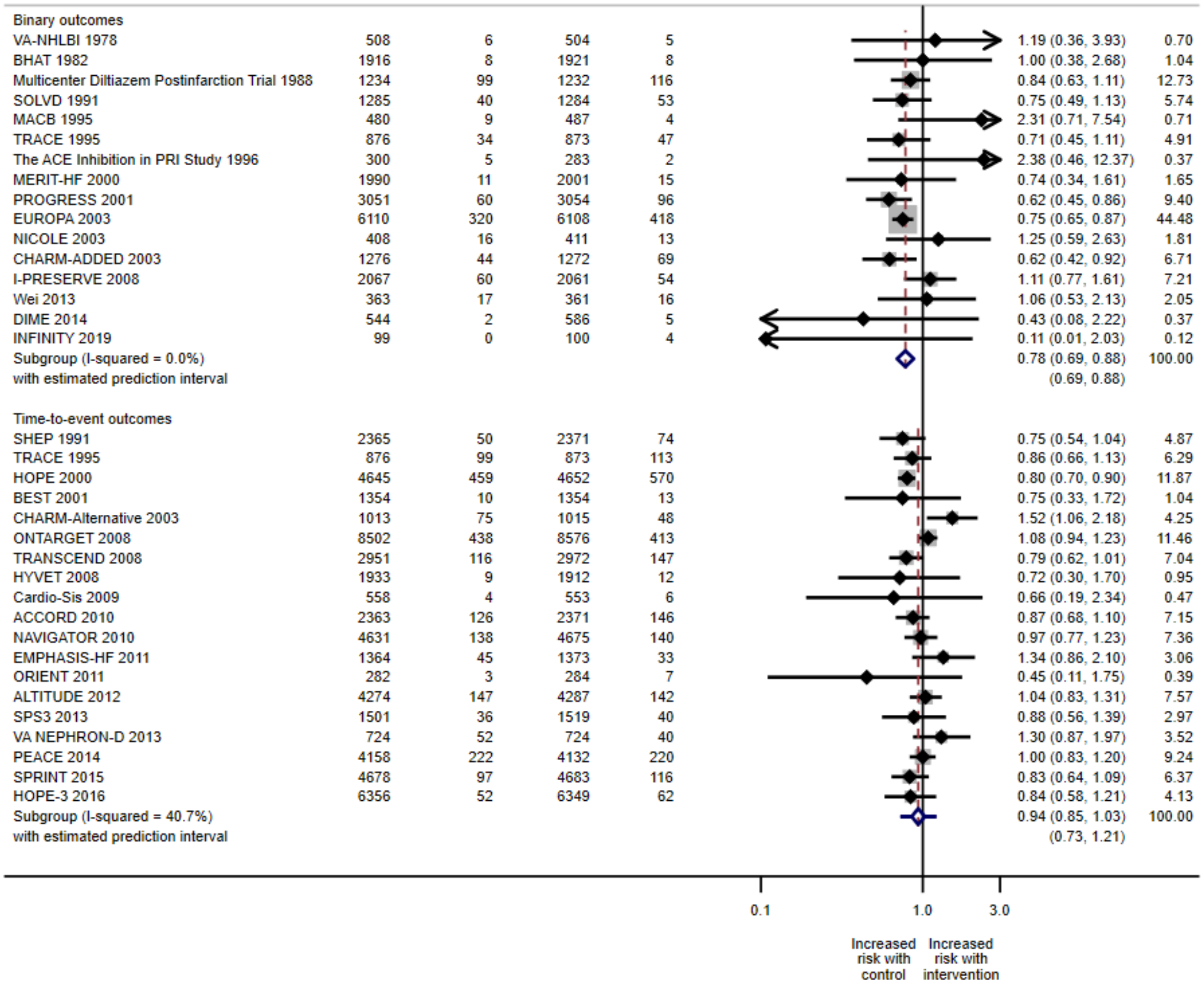

NOTE: Weights are from random-effects model

Supplementary table 3. Meta-regression examining the relationship between the observed treatment effects for each adverse event outcome and study quality

Outcome	Some concerns*		High risk of bias*	
	Coefficient	95% CI	Coefficient	95% CI
Falls	-0.21	(-0.65 to 0.23)	-0.26	(-0.63 to 0.11)
Acute kidney injury	0.38	(0.10 to 0.65)	-0.04	(-0.53 to 0.45)
Fracture	0.75	(-1.75 to 3.24)	-0.11	(-1.87 to 1.66)
Gout	1.96	(-2.77 to 6.68)	-0.08	(-5.71 to 5.54)
Hyperkalaemia	-0.32	(-0.88 to 0.24)	-0.67	(-1.17 to -0.18)
Hypokalaemia	1.13	(-1.07 to 3.32)	-0.30	(-2.56 to 1.95)
Hypotension	-0.20	(-0.66 to 0.26)	-0.14	(-0.65 to 0.38)
Syncope	-0.03	(-0.65 to 0.58)	0.00	(-0.11 to 0.70)

*Reference category – low risk of bias

Supplementary figure 18. Funnel plots showing publication bias in studies reporting acute kidney injury outcomes

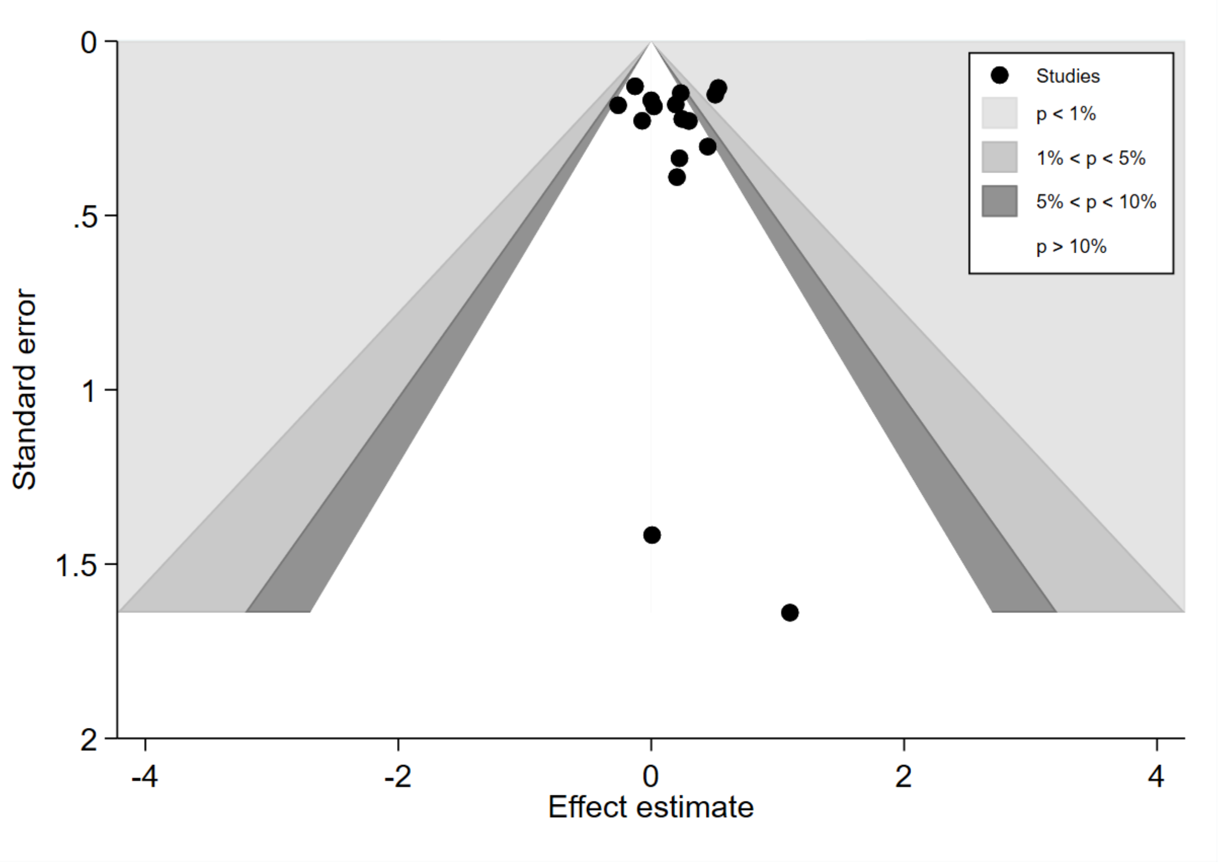

Supplementary figure 19. Funnel plots showing publication bias in studies reporting hyperkalaemia outcomes

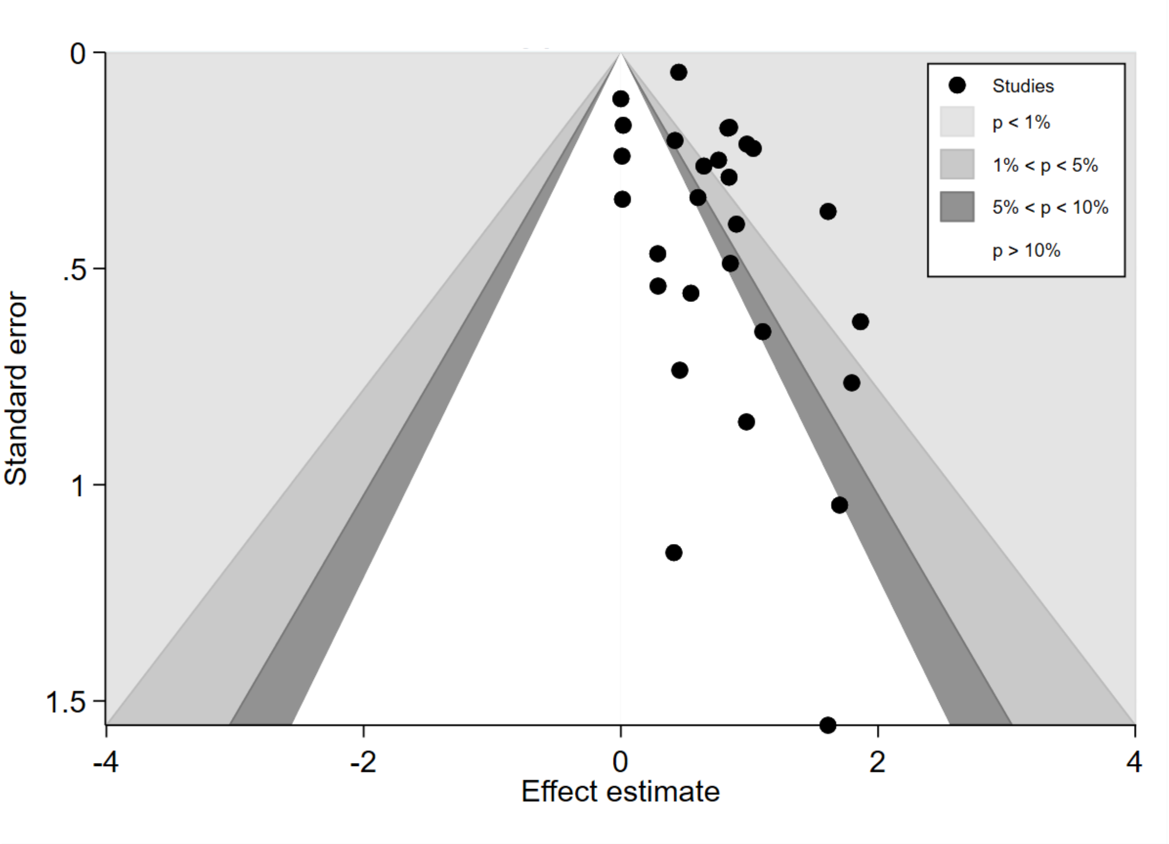

Supplementary figure 20. Funnel plots showing publication bias in studies reporting hypokalaemia outcomes

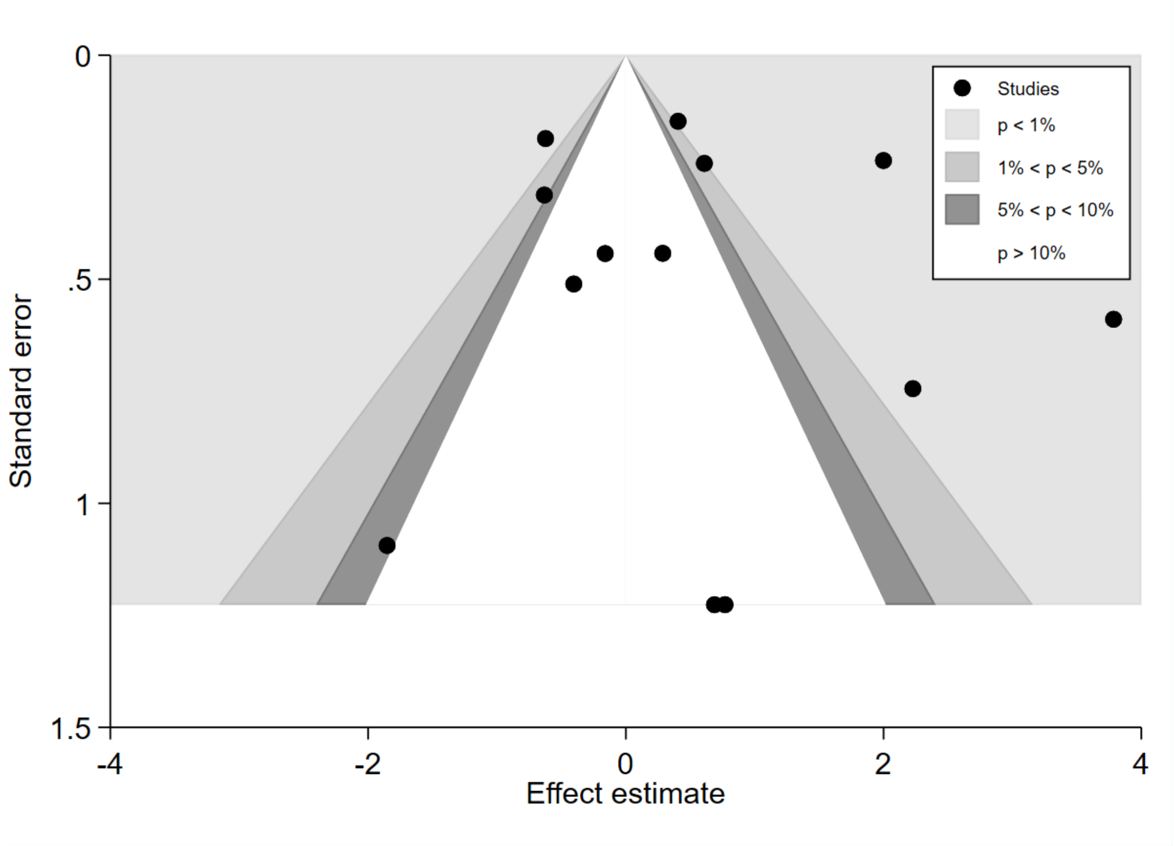

Supplementary figure 21. Funnel plots showing publication bias in studies reporting hypotension outcomes

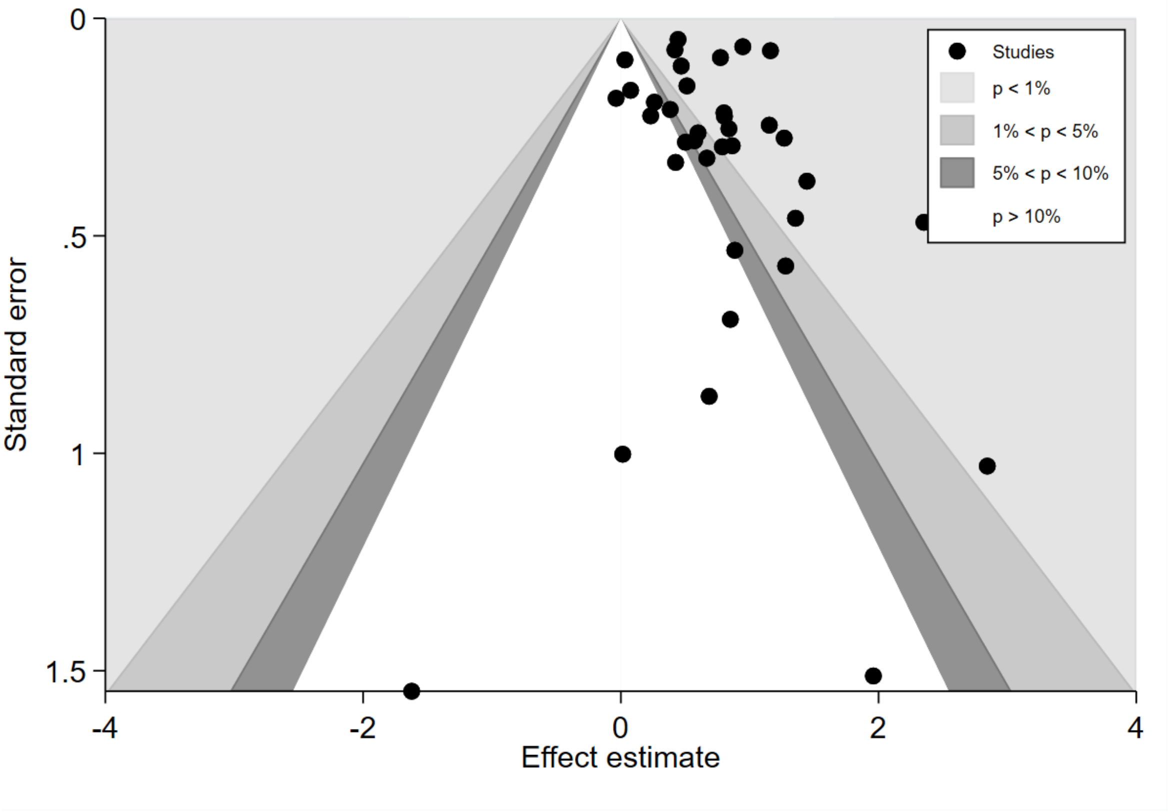

Supplementary figure 22. Funnel plots showing publication bias in studies reporting syncope outcomes

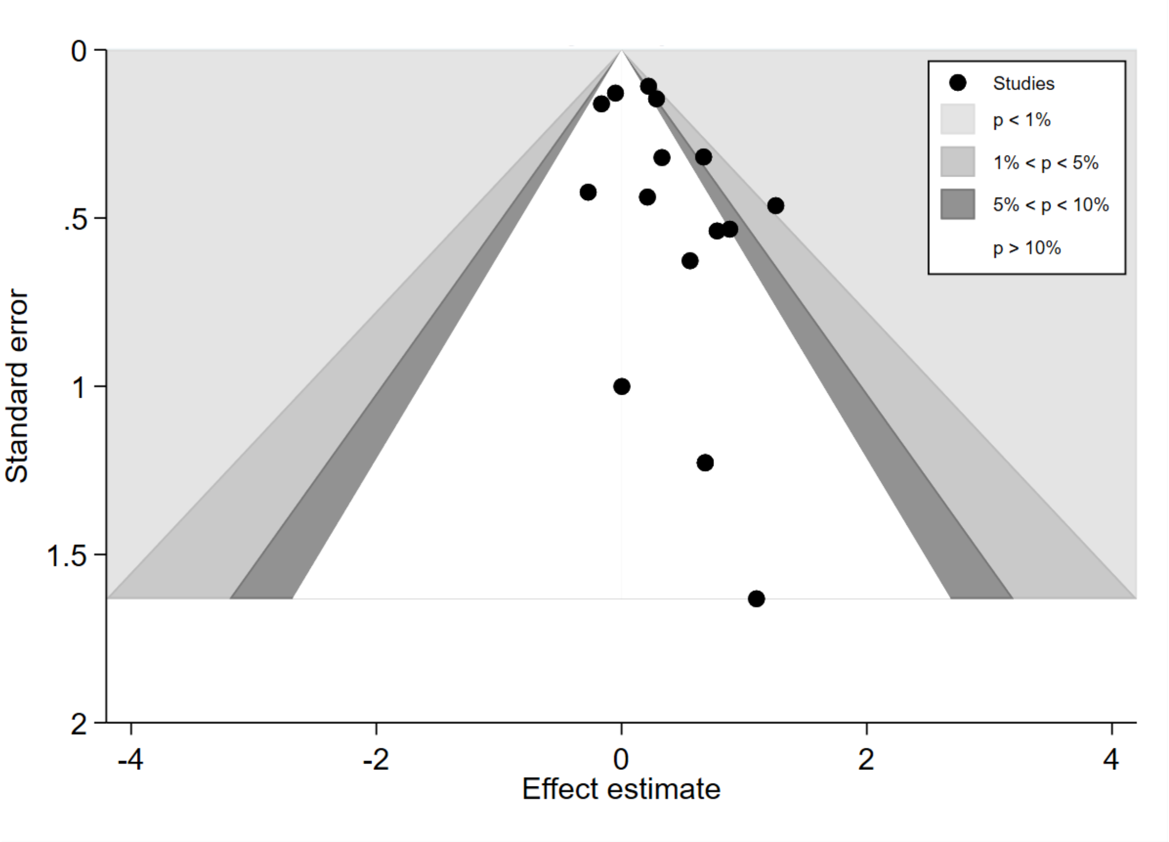

Supplementary figure 23. Random effects meta-analysis of randomised controlled trials examining the association between antihypertensive treatment and acute kidney injury leading to permanent withdrawal from each trial

Study name	Total in intervention group	No. events in intervention group	Total in control group	No. events in control group	Odds Ratio (95% CI)	% Weight
PRoFRESS 2008	10146	28	10186	18	1.56 (0.86, 2.83)	16.79
ORIENT 2011	282	1	284	1	1.01 (0.06, 16.18)	0.76
EMPHASIS-HF 2011	1360	4	1369	6	0.67 (0.19, 2.38)	3.67
ONTARGET 2008 Combination vs Ramipril only	4251	32	8576	48	1.35 (0.86, 2.11)	29.28
ONTARGET 2008 Combination vs Telmisartan only	4251	33	8542	52	1.28 (0.82, 1.98)	30.78
ALTITUDE 2012	4272	10	4285	5	2.01 (0.69, 5.88)	5.11
EUROPA 2003	6110	20	6108	16	1.25 (0.65, 2.42)	13.61
Overall (I-squared = 0.0%)					1.34 (0.99, 1.81)	100.00
with estimated prediction interval					(0.97, 1.84)	

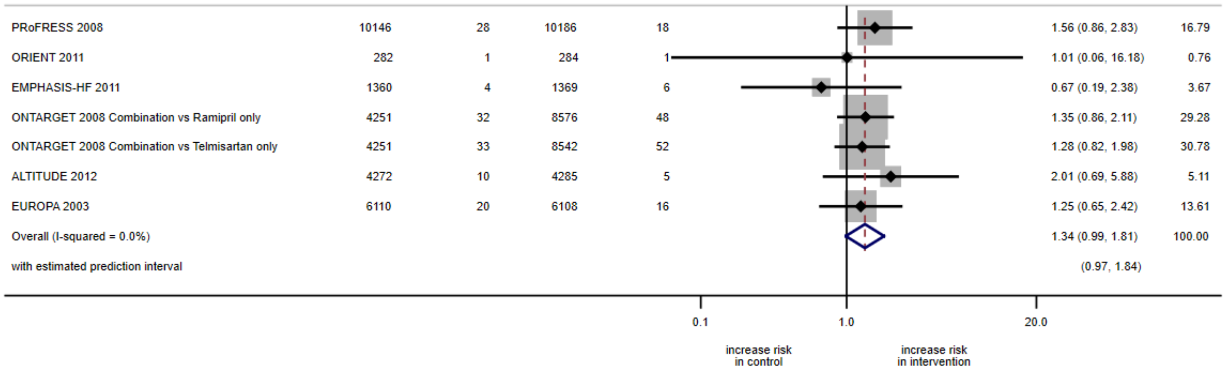

NOTE: Weights are from random-effects model

Supplementary figure 24. Random effects meta-analysis of randomised controlled trials examining the association between antihypertensive treatment and gout leading to permanent withdrawal from each trial

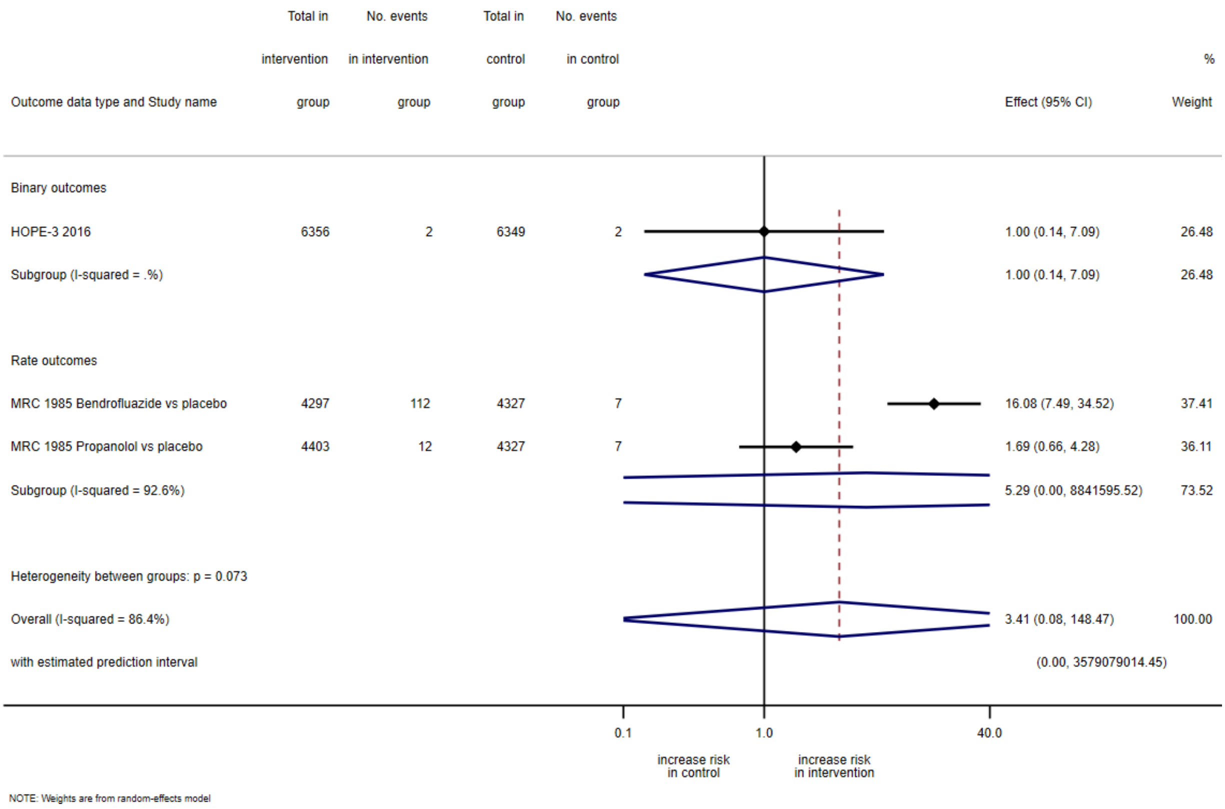

Supplementary figure 25. Random effects meta-analysis of randomised controlled trials examining the association between antihypertensive treatment and hyperkalaemia leading to permanent withdrawal from each trial

Name Year	Total intervention group	No. events intervention group	Total control group	No. events control group	Odds Ratio (95% CI)	% Weight
CONSENSUS II 1992	3044	12	3046	2	6.02 (1.35, 26.94)	2.88
The ACE Inhibition in PRI Study 1996	300	5	283	3	1.58 (0.37, 6.68)	3.09
IDNT 2001 Amlodipine vs placebo	565	3	284	1	1.51 (0.16, 14.59)	1.32
IDNT 2001 Irbesartan vs placebo	579	11	284	1	5.48 (0.70, 42.66)	1.60
CHARM 2003	1514	22	1509	9	2.46 (1.13, 5.35)	8.69
CHARM-Alternative 2003	1013	19	1015	3	6.45 (1.90, 21.86)	4.16
CHARM-ADDED 2003	1276	44	1272	9	5.01 (2.44, 10.31)	9.73
VALIANT 2003 Combination vs Captopril only	2431	6	4879	4	3.02 (0.85, 10.70)	3.90
VALIANT 2003 Combination vs Valsartan only	2431	6	4885	7	1.72 (0.58, 5.14)	5.05
HOPE 2007	4645	8	4652	6	1.34 (0.46, 3.85)	5.31
PRoFRESS 2008	10146	14	10186	6	2.34 (0.90, 6.10)	6.30
GISSI-AF 2009	722	2	720	0	5.01 (0.24, 104.68)	0.75
ORIENT 2011	282	26	284	15	1.82 (0.94, 3.52)	11.06
EMPHASIS-HF 2011	1360	15	1369	12	1.26 (0.59, 2.70)	8.97
ALTITUDE 2012	4272	205	4285	111	1.90 (1.50, 2.40)	27.19
Overall (I-squared = 22.8%)					2.28 (1.70, 3.05)	100.00
with estimated prediction interval					(1.28, 4.06)	

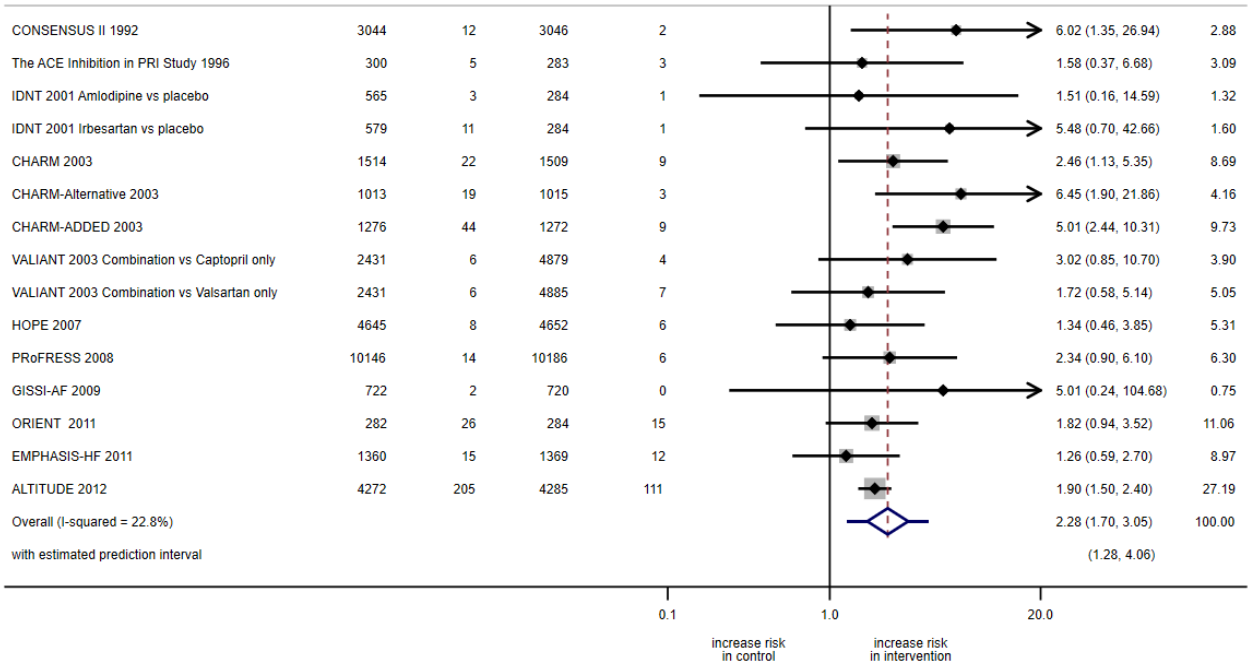

NOTE: Weights are from random-effects model; continuity correction applied to studies with zero cells

Supplementary figure 26. Random effects meta-analysis of randomised controlled trials examining the association between antihypertensive treatment and hypotension leading to permanent withdrawal from each trial

Study name	Total intervention group	No. events intervention group	Total control group	No. events control group	Odds Ratio (95% CI)	% Weight
The Norwegian Multicenter Study 1981	945	26	939	11	2.39 (1.17, 4.86)	3.86
BHAT 1982	1916	23	1921	6	3.88 (1.58, 9.54)	2.60
CONSENSUS II 1992	3044	100	3046	47	2.17 (1.53, 3.08)	9.73
MACB 1995	480	2	487	2	1.01 (0.14, 7.23)	0.61
MERIT-HF 2000	1990	12	2001	5	2.42 (0.85, 6.89)	2.00
PROGRESS 2001	3051	64	3054	29	2.23 (1.44, 3.48)	7.55
Val-HeFT 2001	2511	33	2499	20	1.65 (0.94, 2.88)	5.55
CHARM-preserved 2003	1514	37	1509	17	2.20 (1.23, 3.92)	5.27
EUROPA 2003	6110	60	6108	17	3.55 (2.07, 6.10)	5.82
CHARM-Alternative 2003	1013	37	1015	9	4.24 (2.03, 8.83)	3.66
CHARM-ADDED 2003	1276	58	1272	40	1.47 (0.97, 2.21)	8.23
VALIANT 2003 Combination vs Captopril	2431	45	4879	41	2.23 (1.45, 3.41)	7.88
VALIANT 2003 Combination vs Valsartan	2431	45	4885	70	1.30 (0.89, 1.89)	9.01
PRoFRESS 2008	10146	393	10186	186	2.17 (1.82, 2.58)	15.30
GISSI-AF 2009	722	7	720	3	2.34 (0.60, 9.08)	1.24
ORIENT 2011	282	3	284	0	7.10 (0.37, 137.41)	0.27
EMPHASIS-HF 2011	1360	0	1369	3	0.14 (0.01, 2.78)	0.27
ALTITUDE 2012	4272	28	4285	13	2.17 (1.12, 4.19)	4.34
ADVANCE 2014	5569	69	5571	22	3.16 (1.96, 5.12)	6.79
Overall (I-squared = 34.0%)					2.18 (1.84, 2.58)	100.00
with estimated prediction interval					(1.43, 3.32)	

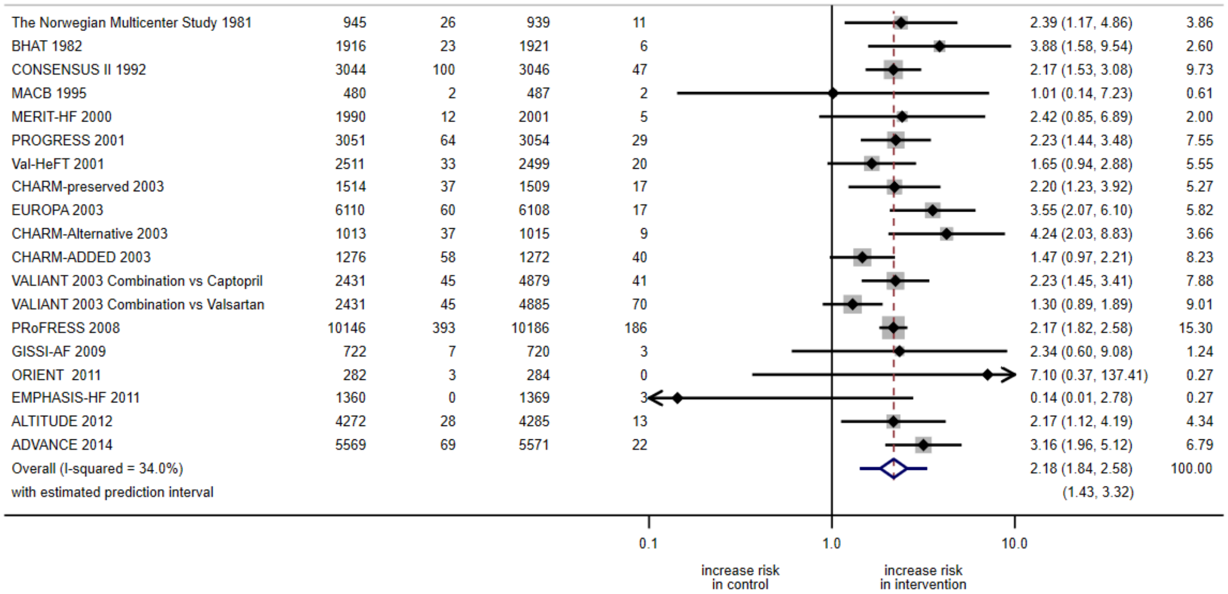

NOTE: Weights are from random-effects model; continuity correction applied to studies with zero cells

Supplementary figure 27. Random effects meta-analysis of randomised controlled trials examining the association between antihypertensive treatment and syncope leading to permanent withdrawal from each trial

Name Year	Total in intervention group	No. events in intervention group	Total in control group	No. events in control group	Odds Ratio (95% CI)	% Weight
HOPE-3 2016	6356	7	6349	4	1.75 (0.51, 5.98)	13.31
PRoFRESS 2008	10146	21	10186	6	3.52 (1.42, 8.72)	24.40
TRANSCEND 2008	2954	1	2972	0	3.01 (0.12, 73.61)	1.97
ONTARGET 2008	8502	29	8576	15	1.95 (1.05, 3.65)	51.61
ALTITUDE 2012	4272	2	4285	1	2.01 (0.18, 22.14)	3.49
BHAT 1982	1916	2	1921	2	1.00 (0.14, 7.12)	5.23
Overall (I-squared = 0.0%)					2.17 (1.20, 3.90)	100.00
with estimated prediction interval					(1.15, 4.09)	

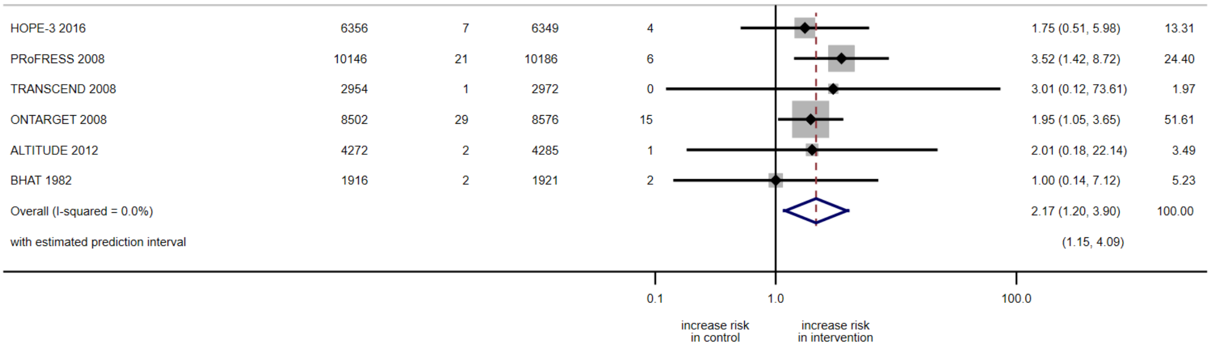

NOTE: Weights are from random-effects model; continuity correction applied to studies with zero cells
